# Co‐Designing Aphasia Services: Evaluation of Involvement and Processes to Support Inclusion of People With Post Stroke Aphasia

**DOI:** 10.1111/hex.70372

**Published:** 2025-08-10

**Authors:** Lisa Anemaat, David A. Copland, Victoria J. Palmer, Sarah J. Wallace

**Affiliations:** ^1^ Queensland Aphasia Research Centre, School of Health and Rehabilitation Sciences The University of Queensland Brisbane Australia; ^2^ Surgical Treatment and Rehabilitation Service (STARS) Education and Research Alliance The University of Queensland and Metro North Health Brisbane Australia; ^3^ The ALIVE National Centre for Mental Health Research Translation The University of Melbourne Melbourne Australia

**Keywords:** aphasia, CCI/PPI, codesign/coproduction, lived‐experience, personcentred care, stroke

## Abstract

**Background:**

People with aphasia (PWA, impaired language/communication) are often excluded from research that concerns them due to a lack of methodological adaptations to support communication. This paper describes adaptations to support their involvement in experience‐based codesign (EBCD).

**Aims:**

To describe the involvement of PWA in EBCD and critically evaluate adaptations required to support involvement.

**Methods:**

Mixed methods process evaluation and reflexive critical appraisal with PWA, significant others (SO), and speech pathologists (SP). Using surveys, stakeholders (*n* = 127) and a consumer advisory group (CAG; *n* = 6) provided feedback on involvement in five stages of the research: (1) online interviews and focus groups; (2) online surveys; (3) consensus meetings; (4) codesign workshops; and (5) the CAG. Critical reflections (lead researcher) informed the analysis. Descriptive statistics and inductive content analysis were used.

**Results:**

Most PWA (79%) liked sharing their experiences online, and contributed as much as desired in group meetings (64%). A modified touchpoint film approach (use of voice actors, still images, and subtitles) supported reflexive discussions and collaborative understanding. All PWA and SO, and most SPs (78%) thought the touchpoint film helped them to understand experiences of care and areas for change. Long‐term engagement in the project was perceived to help build relationships, reduce hierarchical power differentials and support equal sharing of ideas.

**Conclusions:**

Meaningful involvement of PWA was supported through long‐term engagement, a modified touchpoint film approach, and hybrid methods of data collection. EBCD is a suitable approach for exploring experiences of care, identifying leading priorities, and co‐designing areas for change with PWA.

**Patient or Public Contribution:**

This evaluation is informed by the reflections of the research team. This team included the consumer advisory group (public involvement team members) comprising PWA (*n* = 3), SOs (*n* = 2) and a Cultural Capability Officer. Research team members (LNA, DAC, VJP, and SJW) designed the study (including research questions, data analysis processes and assessment measures). Public involvement guided study procedures and recruitment (e.g., methods for engaging with people with lived experience of aphasia), and the interpretation and dissemination of results (e.g., co‐authors on papers). A Cultural Capability Officer advised on culturally safe practices for Aboriginal and Torres Strait Islander participants. Aboriginal and Torres Strait Islander Peoples, are the First Nations people of Australia. Cultural Capability Officer support refers to the support provided to ensure behaviours, systems and processes are conducted in a way that is culturally respectful. Research is reported in line with the GRIPP‐2 guidelines for reporting patient and public involvement.

## 1 Introduction

Participatory approaches such as codesign or coproduction recognise ‘patients’ as *experts* of their lived experiences and draw on this expertise to design solutions based on needs [1]. In the health and disability sector, additional support is often required to ensure meaningful, lived‐experience codesign takes place. However, inclusive practices for these populations within codesign processes are often not fully understood or readily available [1, 2]. This extends to people with aphasia (PWA), who are often excluded from research due to researcher lack of awareness or responsiveness to communication support needs [3, 4]. Aphasia is an impairment of language (speaking, reading, writing, understanding) that impacts communication, and which occurs most commonly following stroke [5]. Previous research has demonstrated that PWA can be meaningfully included in participatory research (e.g., human centred design [6], co‐reseachers [4, 7, 8], codesign [7, 8, 9, 10, 11, 12, 13]). Modifications to accommodate their inclusion [3, 14, 15, 16] have been described and more recently, frameworks to guide inclusion (e.g., *PWA and Other Layperson Involvement‐PAOLI framework* [13]) have been developed [13].

Our research team conducted the first program of research using experience‐based codesign (EBCD) [17, 18] to codesign aphasia service elements [16]. Building on previous large scale EBCD projects [19], those using inclusive practices with specific populations [1, 20, 21, 22], and those conducted in Australian healthcare environments [20, 23, 24], we adapted the EBCD approach to support inclusion of PWA [16, 25, 26, 27]. We hypothesised that the inclusive and flexible nature of the methods, would accommodate the communication support strategies and modifications needed to facilitate meaningful involvement. Modifications drew on learnings from previous research where the participation and involvement of PWA has been supported in other research designs by reducing cognitive load (e.g., reducing amount and complexity of content) [28], use of supportive communication strategies (e.g., multi‐modal communication techniques, offering fixed choice responses, or confirmation of meaning) [29], adapting written information in line with aphasia supportive formatting [30], video recording interviews so meaning can be verified (e.g., non‐verbal communication) [31], and scaffolding codesign techniques (e.g., creative open discussion, approaches to think from others' perspectives, visual analogue scales for collaborative decision making, usability and prototype testing) [7, 10, 12]. We also considered the involvement of PWA in the context of common EBCD challenges: (1) managing power relations, (2) methods for gathering experiences (e.g., choice of method influencing depth of understanding of experiences), (3) maintaining commitment to the process (across sites and contributors), (4) designing improvements (involvement in Codesign), and (5) implementing improvements and subsequent impact [24]. However, to our knowledge, a description of adaptations to support inclusion of PWA in EBCD, is yet to be reported.

The aim of this study was therefore, to describe the process of involving PWA in our EBCD studies [16, 25, 26, 27] and to critically evaluate the adaptations required to meaningfully involve people with communication support needs [32].

### 1.1 Overview of Methods Used

**Figure 1:**
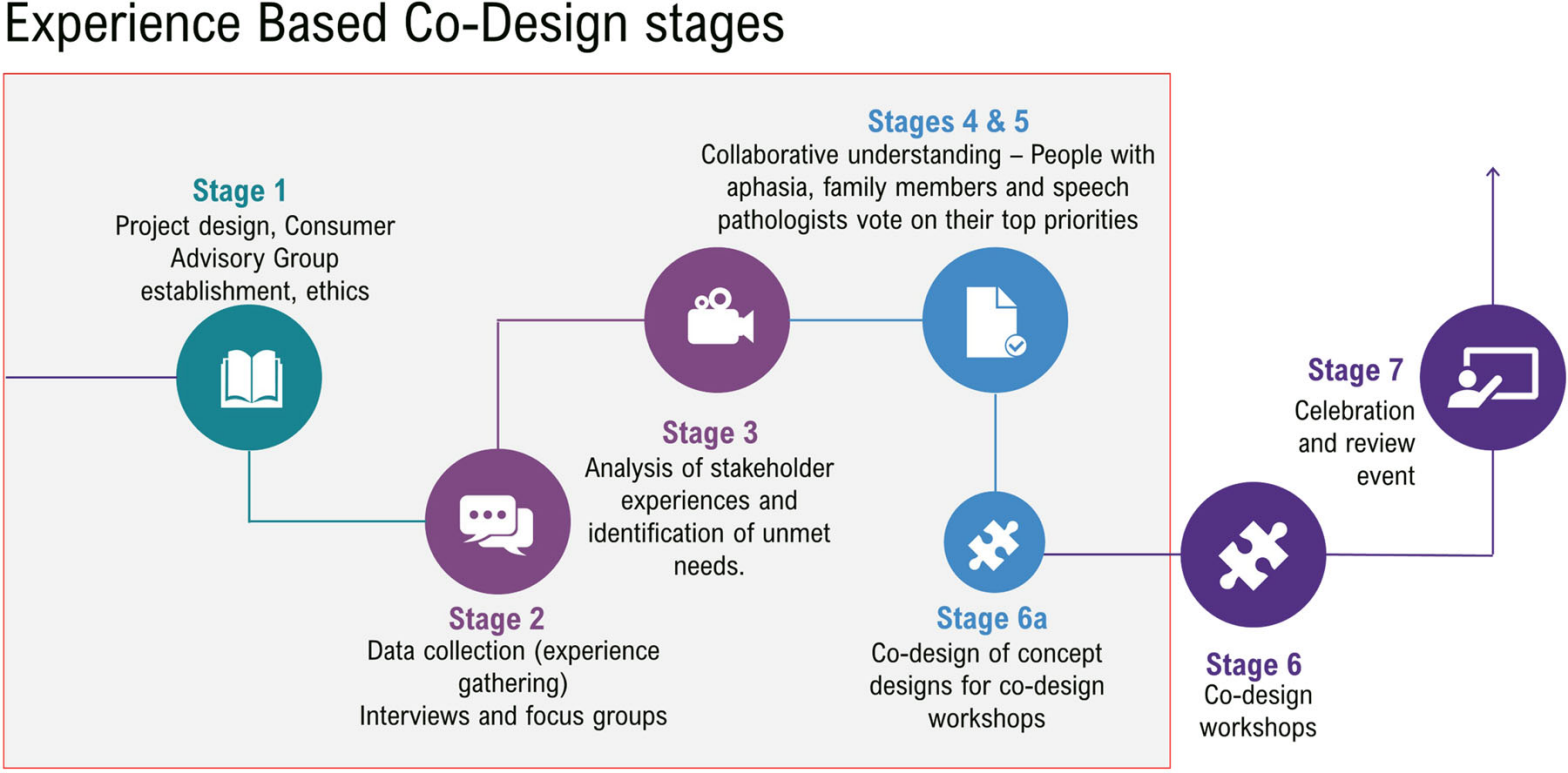
Research overview showing the seven stages within this experience‐based codesign project. Stages appearing in the grey box represent those contributing to data being reported on in this study.

This was an evaluation of an EBCD study (see Figure 1) to Codesign aphasia service elements (e.g., accessibility of treatments, service environments, transitions between services, care provision approaches). Participants were PWA, their significant others (SO), and speech pathologists (SP), from 26 geographically diverse hospital and healthcare sites (hospital‐based and community‐based care) in Queensland, Australia [16, 25, 26, 27]. A consumer advisory group (CAG) contributed to the design of processes and resources across all stages of the research. They were not participants in the study.

#### 1.1.1 Consumer Advisory Group

**Figure 2:**
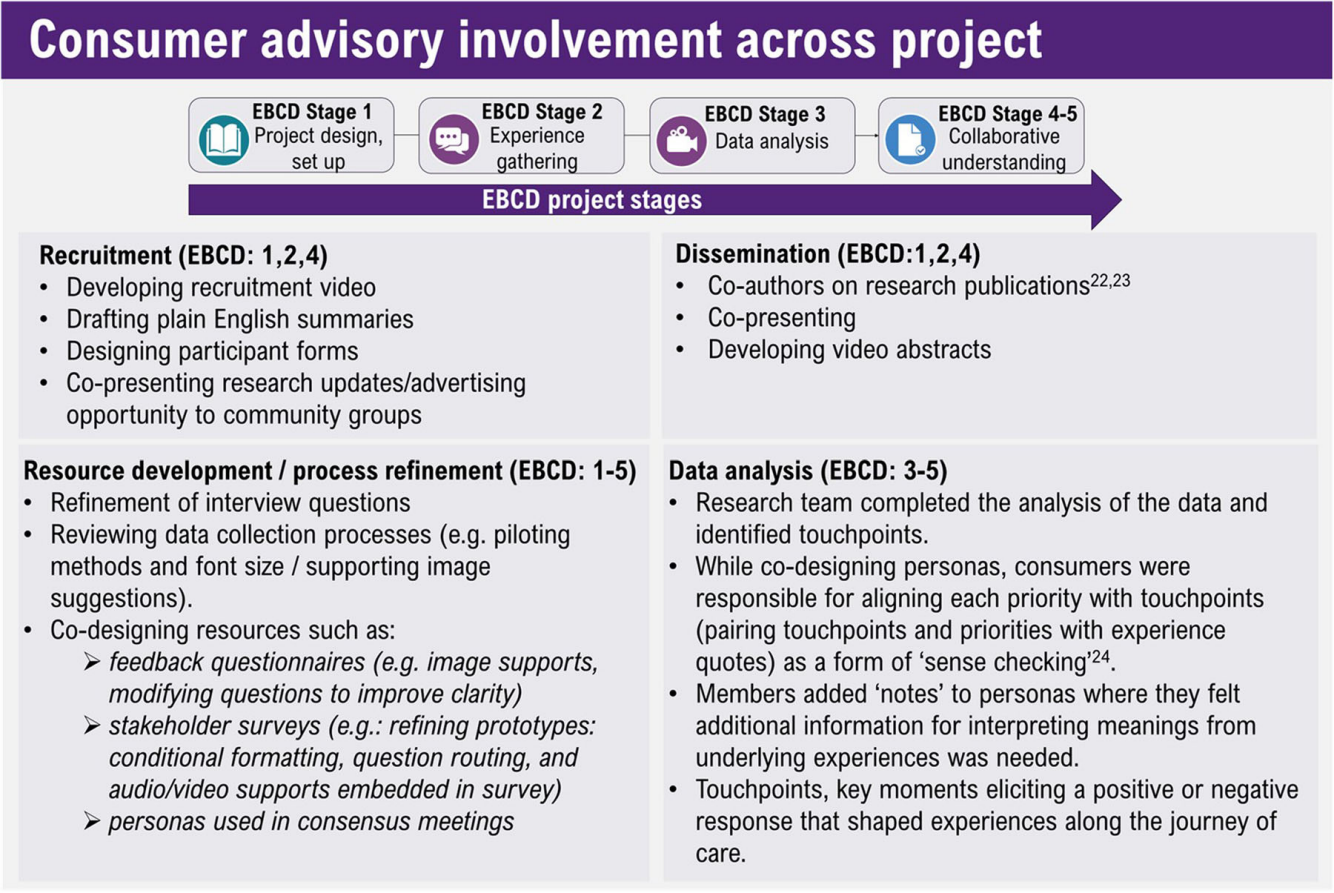
Consumer advisory group involvement across each stage of the project.

A CAG (PWA *n* = 3, SO *n* = 2) provided research oversight and contributed to definition of project boundaries (i.e., types of participant groups and scope of recruitment). Figure 2 provides an overview.

## 2 Methods

We conducted a mixed methods process evaluation (using feedback surveys) and reflexive critical appraisal of our adapted EBCD approach [16, 25, 26, 27]. Data collection occurred during the COVID‐19 pandemic (2021–2022), in the context of physical distancing restrictions. Reimbursement (one‐off gift‐card) was offered to participants, and health services were offered site‐specific reports summarising identified unmet needs and priorities [25, 26, 27]. Appendix describes process evaluation participants. Supporting Information S1: Material S1 provides an overview of participant contributions across research stages.

### 2.1 Process Evaluation

**Figure 3:**
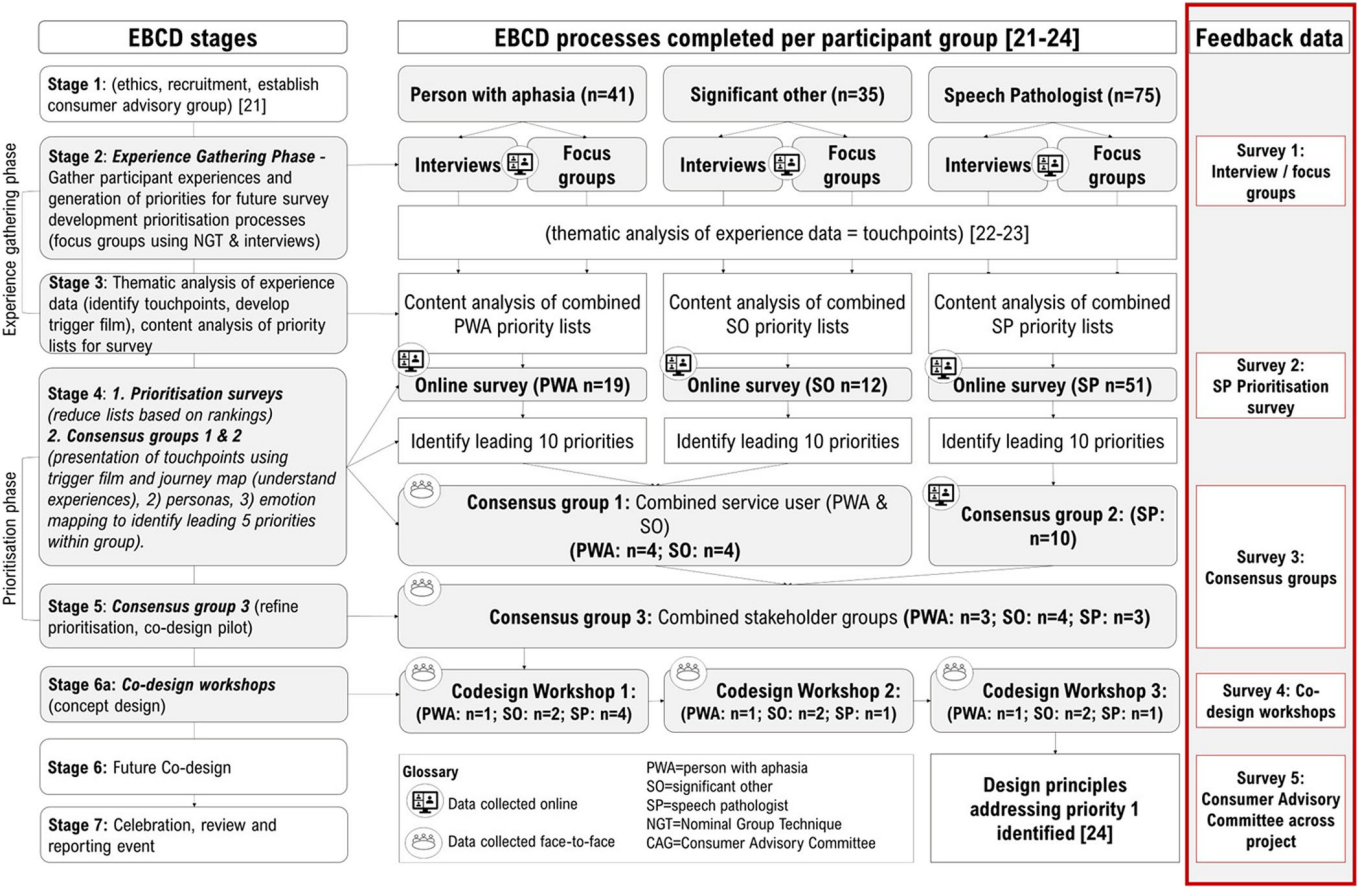
Research overview showing alignment of data collection across each stage of project and where feedback on involvement was gathered.

Each stakeholder group completed a feedback survey per research activity: (a) experience gathering (EBCD‐2); (b) prioritisation (EBCD‐4); (c) consensus groups (EBCD‐4‐5), (d) codesign workshops (EBCD‐6a); and (e) CAG (Figure 3). Surveys conducted following interviews, focus groups and prioritisation activities were modelled on The Point of Care Foundation's EBCD online toolkit [33]. Surveys conducted following codesign workshops and CAG meetings were based on the “Consumer Involvement Evaluation Form” [34]. Data were managed using Excel and analysed using descriptive statistics (frequency counts and percentages). Qualitative data were analysed using inductive qualitative content analysis [35]. Meaning units, coding, and category identification was completed by LA.

- **Experience gathering (EBCD‐2):** (1) online semi‐structured interviews conducted with PWA (*n* = 6), SO (*n* = 7) and SP (*n* = 9); and (2) 39 online focus groups conducted with PWA (*n* = 26), SO (*n* = 23), and SP (*n* = 58) [25, 26]. Feedback survey 1: included 10 questions: eight rated on a 5‐point scale (Supporting Information S1: Material S2 shows an example of formatting for PWA), two open‐ended. PWA and SO verbally responded to questions following their interview/focus group, and the lead‐author transcribed responses. SPs were sent a link to an online survey to complete.
- **Prioritisation (EBCD‐4):** Stakeholder rankings (prioritisation surveys) were collated and tallied to determine the leading 5 (PWA/SO) or 10 (SP) priority areas for consensus meetings [25, 26, 27]. Feedback survey 2 (SP only): asked seven questions: five responding on a 5‐point scale, two open‐ended, participants were emailed link to complete.
- **Consensus groups (EBCD‐4–5):** Three meetings (emotion mapping used to rank priority order): (1) in‐person feedback and prioritisation event with PWA (*n* = 4) and SO (*n* = 4), combined and ranked leading priorities from PWA and SO stakeholder surveys; (2) online event with SP (*n* = 10), ranked SP priorities; and (3) joint in‐person feedback and prioritisation event with PWA (*n* = 3), SO (*n* = 4) and SP (*n* = 3), identified top seven priorities [27]. Feedback survey 3: asked 13 questions: nine rated on a 5‐point scale, four open‐ended. Additional feedback was sought on our modified use of touchpoint films [27] (e.g., restricted film duration, modified voice recordings, still images to represent each touchpoint (emotionally charged themes across the journey of care experiences), and modification of visual content in line with aphasia supportive formatting [30]), personas [27] (e.g., image representing a common theme of experiences paired with overlapping priority areas), and emotion mapping [27] (e.g., placement of sticky‐notes to rank items). See Supporting Information 3 for examples of adaptations. Stakeholders were emailed links to complete.
- **Codesign workshops (EBCD‐6a):** Series of three in‐person codesign workshops were conducted with PWA (*n* = 1), SO (*n* = 2) and SP (*n* = 4). Workshop involvement was limited (*n* < / = 10), with representation across stakeholders, to facilitate engagement [12]. Therefore, only three PWA were invited (2 were unable to attend for logistical reasons). Co‐designers worked together to develop concept designs targeting the top priority [27]. Feedback survey 4: asked nine questions: four rated on a 5‐point scale, five open ended. Stakeholders completed paper‐based forms in‐person following final workshop.
- **CAG feedback**: Six CAG meetings, additional meetups to codesign project resources (prioritisation survey (PWA/SO); touchpoint film; personas), and contributions to papers/presentations. Members were encouraged to consider the entirety of their involvement when responding. Feedback survey 5: as per survey 4.

### 2.2 Reflexive Critique

Reflective practice is applied in clinical practice to look back (reflect) on personal clinical experiences and consider what within an encounter worked well or not so well, and to critically examine (critique) what one might change to improve future practice (learning from experience) [36]. This process, framed by the six steps identified within Gibbs reflective cycle, (description, feeling, evaluation, analysis, conclusions, action plan) [37], was used to explore the alignment of the researchers' personal reflections (recorded during experience gathering, data analysis, and write up by lead author) with the experiences of those involved.

### 2.3 Reflexivity

The first author (L.A., female, S.P.) led the research as part of her doctoral studies and administered the feedback surveys. Therefore, the researcher's relationship with stakeholders may have influenced responses. Regular team debriefings (L.A., D.A.C., V.J.P., S.J.W.) and critical reflections helped maintain a focus on potential biases.

## 3 Results

### 3.1 Process Evaluation (Survey Responses)

#### 3.1.1 Feedback Survey 1: Experience Gathering (EBCD‐2, Interviews/Focus‐Groups)

**Figure 4:**
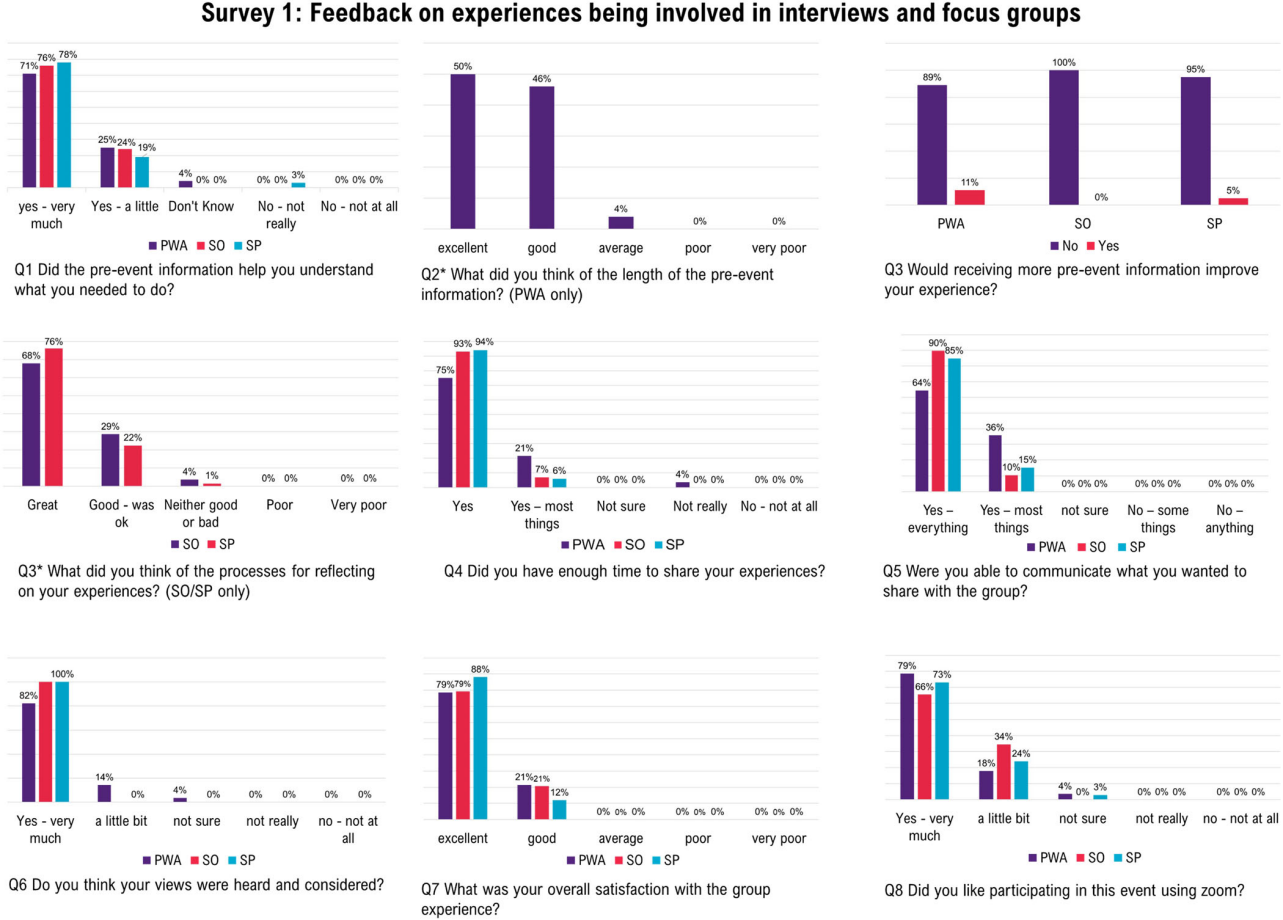
Feedback on experiences of interviews/focus groups (EBCD stage 2) across stakeholder groups. PWA, person with aphasia; SO, significant other; SP, speech pathologist.

A total of 28 PWA (of 32 who completed interviews/focus groups), 29 SO (of 30) and 62 SP (of 67) provided feedback (Figure 4, Appendix, Supporting Information S1: Material S1). Overall, stakeholders expressed satisfaction with interview and focus group experiences. In open‐ended responses about what could be improved, feedback ranged from ‘nothing’ to comments on group size, mode of involvement, processes, group facilitation, group makeup, social connection, and enjoyment with process. PWA commented most often on group size, mode of involvement, or social connection, *“more people, maybe four at the hospital in person. Can look at their expressions”* (PWA‐050), *“It was great that I learnt how to use zoom… really decreased my feelings of loneliness. Very beneficial to your research and to the patients.”* (PWA‐119), *“hmmm… today has been thorough. We can handle it. So today is a good day”* (PWA‐106). Similarly, SOs frequently commented on group size, mode of involvement, or processes, *“small groups – I liked the small group situation”* (SO‐126), *“being more a less a new user* [of zoom] *at this stage I think it was alright”* (SO‐118), *“was good to think about things that have been packed away for a long time”* (SO‐067), and *“it was hard to condense the relevant and important things into a top five”* (SO‐065). SP commented most often on group facilitation, processes, or mode of involvement, *“Great facilitation. Very empathetic listener”* (SP‐054), *“the group ran very smoothly and each person was allocated ample time to share their thoughts, experiences and ideas”* (SP‐074), *“it would be helpful if group members were clearer about sharing one improvement idea at a time… make other group members feel more involved in the discussion”* (SP‐009), *“made me feel comfortable to share ideas”* (SP‐007), “*Always better in person but a Zoom link is much more practical and accessible!*” (SP‐058). While others commented on group makeup or enjoyment with process, *“maybe run groups with clinicians from similar fields. This worked well but I felt there was an acute/community divide”* (SP‐024), *“was difficult to find the time during work hours… however, it was definitely worth finding the time for!”* (SP‐044), *“I enjoyed the collaboration at the end to discuss healthcare/aphasia care priorities”* (SP‐096), and “*larger group‐maybe 4‐5…*” (SP‐047).

#### 3.1.2 Feedback Survey 2: Prioritisation (EBCD 4)

**Figure 5:**
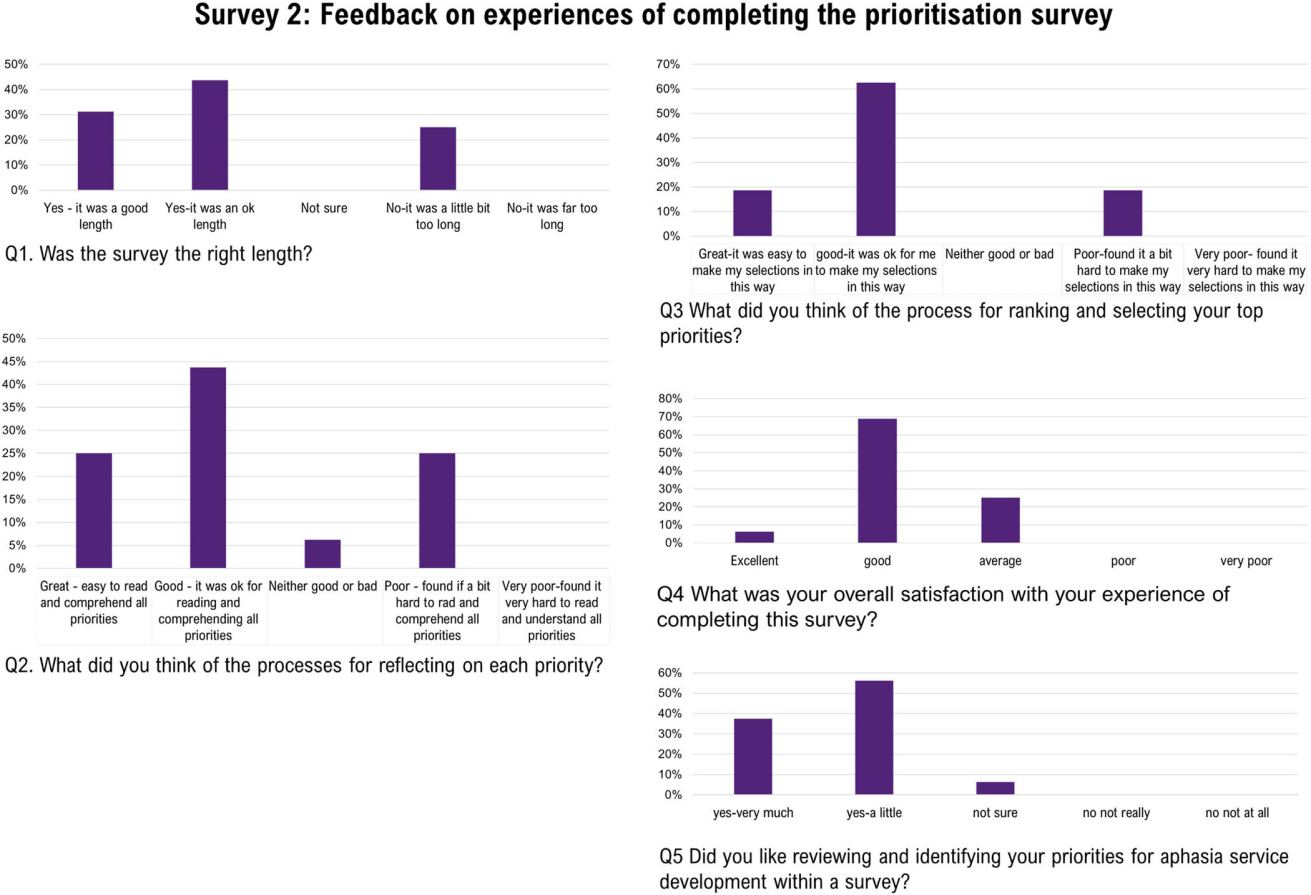
Overview of speech pathologists feedback on experiences of completing the prioritisation survey. Speech pathologist (*n* = 16).

A total of 16 SP (of 51 who completed the prioritisation survey) provided feedback (Figure 5). Nine participants provided responses to open‐ended questions. The majority of feedback related to either on the challenge associated with choosing between 36 items: “*Survey design was great, however I did find it a bit challenging to select my top 5 priorities… as there are so many”* or expressing appreciation for the opportunity to be involved, *“I really liked the opportunity to identify priorities”*. Some responses indicated that the length of items (prioritisation survey) were too long. Feedback was not sought from PWA or SO, however, one SO provided written feedback on behalf of their SO with severe aphasia (completing a paper‐based survey). Within their feedback, they commented that the decision‐making process within the survey was too complex for a PWA and that it took too much time to do this well. Additionally, they expressed concern over other PWA being able to complete the survey independently.

#### 3.1.3 Feedback Survey 3: Consensus Groups (EBCD 4‐5)

**Figure 6:**
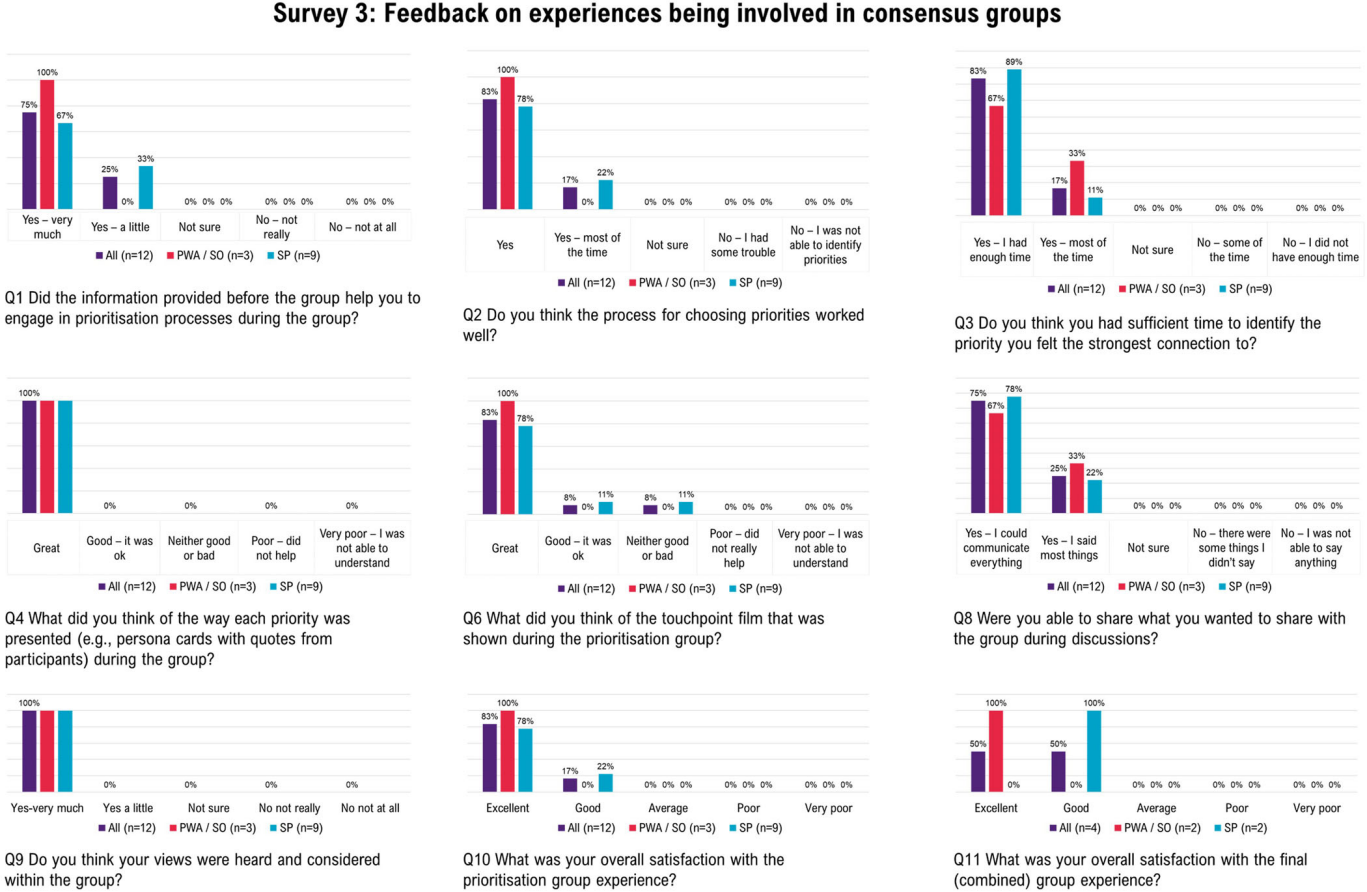
Overview of feedback provided across processes used in consensus groups. Number of individuals contributing to each question is provided. PWA, person with aphasia; SO, significant other; SP, speech pathologist.

A total of 18 stakeholders participated (PWA *n* = 4; SO *n* = 4; SP *n* = 10) and of these 12 provided feedback (PWA *n* = 1; SO *n* = 2; SP *n* = 9) (Figure 6). In response to being asked about the use of emotion mapping, participants across stakeholder groups, unanimously supported this approach for identifying key priorities. Of the open‐ended feedback provided, participants commented: *“very well prepared”* (PWA), *“Great effort on material presented”* (SO‐1), *“I enjoyed the process thoroughly”* (SO‐2), *“seemed very effective and easy to follow”* (SP‐4), *“I personally didn't connect to the persona but like that this was included alongside a number… I liked that each priority was clearly defined with supporting quotes, as this helped me to understand precisely what the priority related to”* (SP‐6). Of those who provided additional feedback in relation to the touchpoint film, feedback included: *“I enjoyed the discussion between fellow members of the group”* (SO‐2), *“were excellent, with simple explanation, clearly set out and moderated speech to assist as wide an audience as possible”* (SO‐1), *“very professional and clear”* (SP‐4), *“*[the touchpoint film] *was interesting in terms of hearing from some of the concerns patients and carers reported. For me it didn't influence how I voted”* (SP‐6). Travel restrictions in response to the COVID‐19 pandemic prevented those from regional or remote areas attending. This limitation was also reflected in feedback, *“I was conscious during the final prioritisations that only people living in the metropolitan area attended”* (SP‐6), *“I think it was important for the PWA & SO to have the face‐to‐face group. … I wonder if we needed more PWA/SO representation from rural/regional Queensland however”* (SP‐8).

#### 3.1.4 Feedback Survey 4: Codesign Workshops (EBCD 6A)

**Figure 7:**
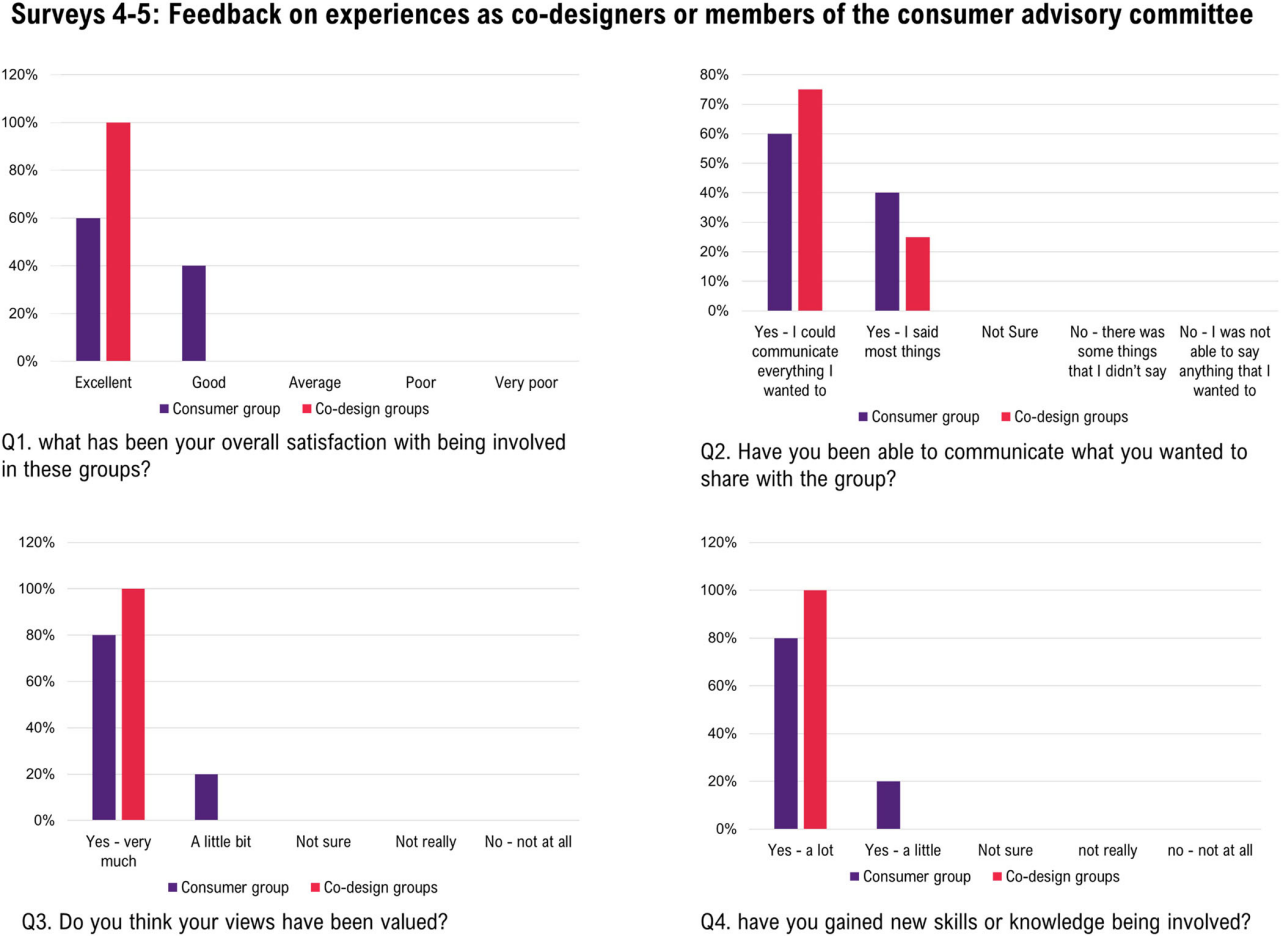
Overview of feedback across experiences being involved in codesign workshops or as members. of the consumer advisory group. Co‐designer feedback: People with aphasia (*n* = 1), significant others (*n* = 2), speech pathologist (*n* = 1). Consumer group feedback: People with aphasia (*n* = 3), significant other (*n* = 2), cultural capability (*n* = 1).

A total of 7 stakeholders participated (PWA *n* = 1; SO *n* = 2; SP *n* = 4) and of these 4 provided feedback (PWA *n* = 1; SO *n* = 2; SP *n* = 1) (Figure 7). All respondents expressed that their views were valued, that they gained new knowledge, and enjoyed their experiences (Figure 7). This is also reflected in their continued involvement (2021–2022), during the challenges of a pandemic, and evidenced in one SP from a regional area taking annual leave to attend. In response to being asked what worked well, *“everything – especially the groups* [Codesign workshops]*”* (SO‐1), *“communication and clarification, prioritising”* (PWA‐1), *“all of it”* (SO‐2), “[facilitator] *being so open to consumer suggestions/changes”* (SP‐1). In response to being asked to describe any challenges or ways Codesign workshops could be improved, *“a lot to take in – but don't stop!”* (PWA‐1), *“more of them* [Codesign workshops]*”* (SO‐1), *“more groups”* (PWA‐1), *“not sure”* (SP‐1).

#### 3.1.5 Feedback Survey 5: Consumer Advisory Group

The CAG consisted of six members (PWA *n* = 3; SO *n* = 2; Cultural Capability *n* = 1) with six providing feedback, (Figure 7). All reported their views were valued, and that they gained new knowledge because of their involvement (Figure 7). What they enjoyed the most varied: *“meeting others in person and something to work at together”* (CAG‐1), *“comradery with the group”* (CAG‐2), *“being able to contribute to a project that will make a difference in the future for PWA”* (CAG‐3), *“learning from PWA and SO”* (CAG‐5), *“group and talking”* (CAG‐5). In terms of what could be improved members commented, *“sometimes meetings were a bit long”* (CAG‐2), *“I think everything was done well”* (CAG‐3), *“conducting research at rural and remote locations”* (CAG‐4).

### 3.2 Reflective Critique of Processes and Methods Used

A reflexive critique of methods and adaptations is provided in reference to five common challenges identified in prior EBCD projects: (1) *managing power relations*, (2) *methods for gathering experiences*, (3) *maintaining commitment to the process*, (4) *designing improvements*, (5) *implementing improvements and subsequent impact* [24]. Table 1 summarises ways to address these challenges, and modifications to support communication when conducting EBCD with PWA.

**Table 1:** How challenges identified by Dimopoulos‐Bick et al., (2018) were addressed at each stage of this EBCD project and suggested modifications.

Stage	Challenge [23]	Planned modifications based on literature	Modifications made
Stages 2, 4–6	**“** ***Managing power relations*** **”**	Five measures were built into our design to help address this: (1) use of deidentified personas; (2) separate initial stakeholder consensus meetings; (3) use of surveys for reducing priority lists; (4) offering reimbursement; (5) codesign training. To help reduce power dynamic influencing voting procedures, and the challenges associated with voting on an individual's experience, deidentified personas will be used for voting. To encourage engagement, participants will rank priority improvement areas within stakeholder groups initially, to refine priority lists before joint prioritisation. Use of prioritisation surveys (allowing those uncomfortable with group voting procedures to indicate their preferences). Providing reimbursement for patients and carers facilitates valuing their input as equal partners. All participants attending codesign workshops will attend codesign training. Training will be informed by codesign pilot and feedback from participant responses on their experiences of being involved in earlier stages. Training has been identified as a useful tool for managing expectations and establishing collaborative codesign groups [23].	Co‐designed personas for representing priority improvement areas Separate (initial feedback) stakeholder groups Hybrid survey approaches to reduce initial lists (based on rankings where list length needs to be reduced) Reimbursement vouchers (including parking vouchers and refreshments for all in‐person sessions) was provided (meetings included time for lunch allowing time for connection) Codesign training/managing expectations (explanation of roles, purpose of activities, and contributions sought, provided at the beginning of each session, and sent out as pre‐reading) Emotion mapping for identifying leading priority improvement areas
Stages 2, 4–5	**“** ***Methods for gathering experiences*** **”** *(use of trigger film and nonparticipant observations are recommended* [29])	Feedback from a previous EBCD project identified that staff felt confronted when viewing trigger (touchpoint) films that evoked strong emotional responses in a combined group setting [23]. To account for this, the touchpoint film will be viewed for the first time within stakeholder groups. This project is considering a system wide evaluation of experiences across services and service pathways that differ considerably. In addition, this project is not evaluating/improving a current service, but rather, is developing a new service that does not yet exist. Therefore, field notes will be collected at each site to provide context to understanding experiences, but in‐depth nonparticipant observations will not be conducted.	Trigger films (designed with communication accessible formatting, links to view sent ahead of meeting – allowing PWA to review content at own pace) Hybrid methods (online/in‐person) options Planning time to include diverse representation Provide training for all data collection/online engagement methods (involvement took place once participant indicated readiness, confidence to engage) All questions provided before involvement (ensuring adequate time to prepare)
Stages 1–2, 4–6	**“** ***Maintaining commitment to the process*** **”**	To combat the challenges associated with reduced management engagement identified in other EBCD projects, this project will conduct nominal groups at each participating site. These consensus meetings will identify priorities based on suggestions for aphasia service development put forward at each location. This will enable the development of tailored reports back to sites for their own quality improvement projects to ensure management buy‐in. Reimbursement for patients and carer involvement will also be provided to acknowledge the investment of time and to cover costs associated with participating.	Offering something in return (value adding, reimbursement) Working with sites to determine suitable times for involvement Offering flexibility in choice of engagement
Stage 6a–6	**“** ***Designing improvements*** **”**	Before codesign groups, participants will undergo training to ensure all members are familiar with the processes and expectations involved and to encourage collaboration between members.	Training for codesign Design principles (scaffolding techniques might include: card sort, prototyping, journey mapping, narrative descriptions, and personas) *Mapping service contexts
Stage 7	**“** ***Implementing improvements and subsequent impact*** **”**	Co‐designed services will be implemented by the Queensland Aphasia Research Centre, all participants will be able to offer feedback on their experiences of being involved across each stage of the EBCD project and on co‐designed services implemented.	Stage not yet completed Use of codesign categories might support understanding of resource commitments

*Note:* 1: Modifications included consideration of: group size/make up, online versus in‐person communication support options. For online involvement: on‐site support for trouble‐shooting device/connection issues and ways to ensure safety (who to call if a PWA became unwell, distressed, or fatigues while sharing emotionally taxing experiences online). 2: For non‐English speaking participants, considerations included: pre discussions of supportive communication strategies with interpreters, translating materials, reviewing culturally safe practices, and translation of interview transcripts. *Mapping involved: (1) describe the journey of care (care pathways) for a PWA (from point of entry to no further service access); (2) divide the journey into 3–5 key stages; (3) identify barriers for PWA accessing care at each key stage; (4) identify what the service is able to do well/not so well per stage; and (5) identify confidence of the team delivering care per stage.

#### 3.2.1 Managing “Power” Relations

Codesign methods encourage the collaboration of multiple stakeholders as equal partners in co‐designing solutions [24]. Managing power between those involved is often described as a challenge for authentic Codesign. This stems from two key areas, traditional patient‐healthcare provider hierarchical relationships, and the vulnerability of sharing lived‐experiences [24]. To address this, five measures were built into our design (Table 1).

In the prioritisation phase (consensus workshops), co‐designed personas [27] removed challenges associated with voting for an individual's experience and ensured a range of experiences were reflected. Separate stakeholder consensus meetings were observed to encourage those involved to share reflections more freely and discuss experiences in relation to touchpoints and experience maps. This is comparable with Dimopoulos‐Bick et al., [24] findings, suggesting separate initial feedback sessions be held. Surveys were a valuable tool for reducing priority lists and involving those from regional and remote areas in prioritisation.

#### 3.2.2 “Methods for Gathering Experiences”

##### 3.2.2.1 Consumer Advisory Group

The CAG played a vital and active role in recruitment, designing and piloting resources and processes, reviewing data interpretations (e.g., pairing experience quotes, unmet needs, and touchpoints) and dissemination of project materials (e.g., co‐authors, co‐presenters on research). Considerations included: (1) time (preparation of meeting agendas/minutes/resources in accessible language formats, co‐creating and piloting project resources/dissemination activities); (2) accessible language formats (see section on supports for PWA below); (3) consistent membership (facilitated relationship building/understanding); (4) meeting to discuss papers, presentations, or pre‐record co‐presentations. Meeting face‐to‐face was observably more productive and the preferred option.

##### 3.2.2.2 Geographic Remoteness

Co‐ordinating with local community centres or peer support groups enabled computer access and support for navigating online involvement for PWA (unfamiliar, or lacking confidence with engaging online). Natural disasters during data collection, added requirements for additional planning (e.g., when to post information and safety for support partners driving to remote areas cut off during flooding). Other considerations included: identifying the stability of internet connectivity, device availability, and posting resources (e.g., visual aids) to ensure comprehension for participants with unstable internet connections or using a phone to dial in. In some cases, members of the research team drove to regional areas to drop off devices and internet dongles.

##### 3.2.2.3 Familiarisation and Training

Training involved posting or emailing instructions for connecting over Zoom, talking through connection processes (via phone), trialling Zoom features, confirming communication supports, and reviewing engagement processes. For those with a severe aphasia, additional support was sought (e.g., one participant worked with their community SP to prepare responses). Familiarisation with online engagement processes, platforms, and research questions, was seen to increase confidence and support engagement.

##### 3.2.2.4 Supports for People With Aphasia

Experience gathering phase: Participants were given the choice of experience sharing modality (interview or group engagement). Sharing in a group while navigating technology was considered too challenging for some who preferred an interview. For others, learning to connect using online methods was a valuable way to relate to peers. Another outcome of long‐term commitment were opportunities to link PWA with longer‐term public involvement activities. During experience gathering sessions, participants shared experiences of care, followed by ideas for improvement. This supported idea generation, whereby shared ideas were observed to build on shared experiences. Personal experiences were drawn on to scaffold communication (e.g., probing questions specific to experiences of care shared were drawn from during idea generation).

Prioritisation phase: Conditional formatting within the online survey limited choice options for PWA, providing support to direct them through the survey, a function not available on the paper‐based version. Supporting Information S1: Material S3 provides details of formatting used to support communication in the online survey. This formatting may have reduced the complexity of navigating the survey for some PWA. Offering an in‐person option could accommodate strategies to ensure comprehension, provide reassurance, and limit stress associated with navigating an online platform. Additionally, consensus groups were held in‐person to support PWA to contribute. A limitation associated with running consensus groups in‐person, was that only those who lived locally (metropolitan area) were able to attend. Further comprehensive descriptions of adaptations to the experience gathering [25] and prioritisation [27] phases have been described elsewhere.

##### 3.2.2.5 Supporting Aboriginal and Torres Strait Islander Peoples

Involvement of the Cultural Capability Officer (CAG member) was considered a strength. Regular access to cultural support and guidance (e.g., critical reflection during regular debriefs, to reflect on nuances of language) supported Aboriginal and Torres Strait Islander Peoples to contribute. While conducting experience gathering processes online was a strength (e.g., supporting diverse involvement across geographic remoteness), it was also a limitation, presenting a barrier to establishing trust or facilitating access.

##### 3.2.2.6 Time and Resource Constraints

Data collection took place during the COVID‐19 pandemic, requiring additional adaptations. Travel and face‐to‐face restrictions, increased community stress (peak periods of COVID‐19 transmission), and reduced capacity of sites to support research, lengthened data collection times. Supporting Information S1: Material S4 provides an overview of involvement timelines. Despite the extenuating circumstances, some learnings might be relevant. For example, we found online group size (limited to *n* = 3) with PWA worked well, and many experienced fatigue beyond 1.5–2 h. This is comparable with research exploring online methods of data collection with people with communication disorders [38].

##### 3.2.2.7 Touchpoint Films

Touchpoint films used to support collaborative understanding of experiences, is considered best practice in EBCD [39]. Touchpoint films typically capture representative footage of stakeholder experiences associated with positive and negative touchpoints, shared in service users own words, and embedded with emotional underpinnings. The touchpoint film developed in this project used still images and voice actors to read representative quotes instead of the initially planned footage from filmed experience data [27, 40] (Supporting Information S1: Material S3). This choice was made for two reasons, the quality of footage captured (online data collection) lacked the quality needed to develop a cohesive film, and to accommodate modifications for PWA (e.g., artificial modulation of voices to maintain a consistent rate of speech and volume). Touchpoint films are used to convey initial interpretations to a wider audience and seek feedback as part of the codesign process [17]. This is to ensure that ‘anti patterns’ or the biases of those interpreting the data are avoided [17]. Facilitated stakeholder reflections on the touchpoint films, supported the representativeness of touchpoints presented, and viewing the film observably generated significant emotional responses in PWA/SO present (who voiced connections with depicted experiences) [27]. This suggests that both the method for developing our touchpoint film, and touchpoint interpretations, were valid.

##### 3.2.2.8 Experience Maps

Visual synthesis tools, such as experience maps, are an effective method for conveying complex information related to healthcare experiences [41], and have been used in prior EBCD projects [23]. Experience maps were developed to demonstrate where defining moments of experienced realities (connecting with services) took place across the continuum of care [25]. Each experience map showed a visual synthesis of the frequency of touchpoints being reported at three key phases of care [25]. Subjectively, the use of experience maps to synthesise the breadth of experience data shared, supported collaborative understanding of experiences.

#### 3.2.3 Maintaining “Commitment to the Process”

##### 3.2.3.1 Speech Pathologists

To ‘give back’, local sites were offered (deidentified) reports summarising key touchpoints and priorities identified by their service users (PWA/SOs) and providers (SP). One challenge with this approach was co‐ordinating schedules for multiple staff working in the same department. An unforeseen benefit to gathering experiences by local areas was expressed by a SP: “*I appreciated how it was grouped by region, much easier to have conversations (1) with others that you know, and (2) about issues etc that are relevant to your location and experience*” (SP‐011). As reported by previous EBCD projects [24], having management on board enhanced engagement.

##### 3.2.3.2 Consumer Advisory Group

As a PhD research project, funding to support consumer involvement was limited. CAG members discussed, identified and agreed how they would like to receive something back. For example, some members supporting local aphasia groups, appreciated the opportunity to hear updates (that they could report back on) or having a SP attend their meetings. Opportunities to copresent on the research and coauthorship were also supported. The ongoing commitment to contribute, speaks to how well the group worked together.

#### 3.2.4 “Designing Improvements”

##### 3.2.4.1 Maintaining a Focus on Experience

Prioritisation processes were adapted to reduce complexity for those voting (*n* = 773 ideas). Priorities were themed and combined to simplify survey content. To ensure focus remained on stakeholder experiences, in subsequent EBCD stages, emotion mapping was used to identify leading priority areas (Supporting Information S1: Material S3). Initial emotion mapping involved PWA/SO placing two different coloured sticky notes (first = persona they felt the strongest emotional connection with, second = persona they felt the least emotional connection with [20]). Following the first round, participants reflected they were not able to select something of ‘less importance’ and considered all priority areas important to someone. Therefore, identification of personas representing their strongest emotional connection only was completed in subsequent rounds. Feedback provided on emotion mapping suggest processes used were effective for identifying top priority areas, focused on emotional connections with experiences.

##### 3.2.4.2 Using Design Principles

In codesign workshops, design rules [42] (or principles) were used to collaboratively synthesise and translate an understanding of experiences into actionable change to address gaps in care [17]. The application of design principles (e.g., if you want to achieve (Y) outcome (or goal) in context (S), then activity (X) might help), is typically used in EBCD projects for refining priorities for service intervention [17, 42]. This collaborative process uses design tools to encourage stakeholders (not experienced in design methods), to think creatively as designers [43]. Co‐designers created personas (pairing an image, name, characteristics of impairment, fears, frustrations, and anxieties) in pairs. Personas were used as a reference point for subsequent activities during codesign workshops. The priority focus was explored in group discussions aimed at reframing the problem, ideas were grouped and themed into actionable goals. Defining the context involved exploring possible challenges for their ‘persona’ within the hospital environment (e.g., what do they want, feel, see, hear, taste, smell). Ideas were generated in small groups before rounds of discussion with the wider group. Determining actions involved: story boarding (describing a solution, how it helps, and who is involved), world café [44] (pairs wrote solutions to problem statements, then passed responses to the next group to add to), and five‐steps (participants are provided with five cards and asked to describe a solution to the problem in five steps, one step per card). This final activity (five steps) while attempted, was too complex to continue with.

Codesign processes were piloted with a small group who had developed relationships over the course of their extended involvement. A personal reflection on the codesign workshops is two‐fold: holding the groups close together supported those involved to attend. However, it also limited time available to modify resources needed to support communication and for PWA to familiarise themselves with content ahead of sessions. Supporting Information S1: Material S5 provides additional detail according to the PAOLI framework [13].

##### 3.2.4.3 Mapping Service Contexts

During interviews, a sample of SPs were asked to map their service context (Table 1, Supporting Information S1: Material S6). This information provided contextual support during analysis and is valuable for informing future codesign work where an understanding of aphasia service pathways in Queensland, Australia is needed.

#### 3.2.5 Implementing Improvements and Subsequent Impact

**Figure 8:**
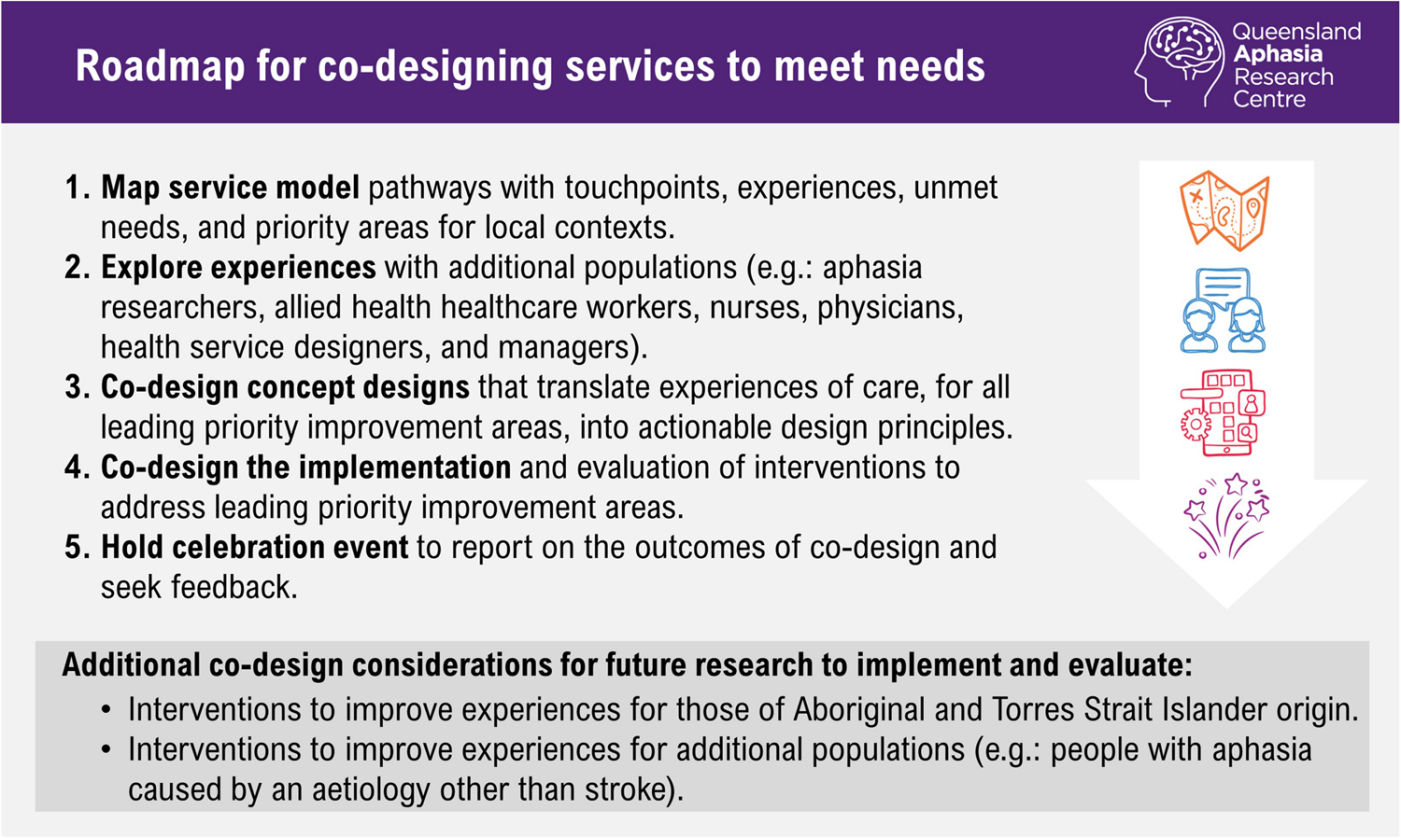
The following steps have been identified for future research aiming to design services to improve experiences for people with aphasia, their significant others, and the speech pathologists providing their care.

Pilot codesign processes revealed multiple design principles targeting one priority and highlighted elements of project scope, required to address needs [27]. Applying codesign categories [20, 45] to design principles identified, may also support resource planning for co‐designing the implementation of solutions. Future implementation efforts (including those planned by this team) should ensure interventions designed to meet needs are suitably scaled for service contexts (Figure 8 shows a roadmap for aphasia service development).

## 4 Discussion

This study evaluates and provides a critical reflection of modifications to EBCD to support the participation of PWA. We identify three new learnings that may contribute to expansions in the EBCD field and facilitate inclusive practices for those with communication support needs. These learnings relate to: (1) the hidden value of longitudinal EBCD projects, (2) hybrid methods of engagement, (3) touchpoint film development. Collaborative research approaches used (e.g., idea generation, joint prioritisation), including modifications that enabled meaningful engagement of PWA, may also be applicable for applying clinically (e.g., collaborative approaches to goal setting [6, 46], planning care [47], or for shared decision making) [48].

### 4.1 Hidden Value of Longitudinal EBCD Projects

We found the long‐term nature of this project (involvement over an extended time frame: May 2021–September 2022), allowed relationships to form between members. Those involved often commented on the value of these relationships, and it was observed that this changed the power dynamic among stakeholders. Stronger relationships amongst members fostered mutual respect for contributions, collaborative engagement during activities, and members gained an understanding of communication strengths and preferences (e.g., when additional processing time or breaks were needed, and regular use of communication support strategies such as writing key words or drawing without facilitator prompts), which facilitated inclusive practices. While sessions began with an overview of the lived expertise each person brought, explanation of activities and expectations, training designed to address and challenge hierarchical power differentials was not required. Addressing power differentials, has previously been identified as a challenge for meaningful codesign, and is often a barrier to inclusive codesign [1, 2]. Whilst there is interest in ‘accelerating’ the sometimes‐lengthy EBCD process, perhaps finding ways for longer‐term relationships between stakeholders to develop, holds value for ensuring inclusive engagement during codesign processes for populations with communication support needs.

### 4.2 Hybrid Methods of Engagement

Modifying processes and supportive strategies enabled meaningful online engagement. This facilitated diverse representation and engagement across geographic areas. However, some PWA commented on the need for a mix of face‐to‐face and online activities to support relationship building. While recent research acknowledges the validity of using online data collection methods with people with communication impairments [38], it also recognises that online involvement can be more (cognitively) demanding, requiring shorter sessions [38]. A perceived lack of access or familiarisation with navigating or communicating using online systems, may have discouraged some people from being involved. For at least three PWA who expressed interest (and desire to attend in person), online involvement prevented participation. Perhaps a hybrid approach, with online and face‐to‐face options for gathering experiences, would support a more diverse sample to be involved. An acknowledged limitation of these findings is the failure to collect feedback from PWA/SO participants contributing to the prioritisation survey, the small sample contributing to codesign workshops, and the lead‐authors role in collecting feedback.

### 4.3 Touchpoint Film Development

In typical EBCD projects, touchpoint (trigger) films have used video clips of stakeholders sharing experiences associated with identified trigger points to facilitate collaborative understanding of experiences. We present a potential alternative method for developing touchpoint films (Supporting Information S1: Material S3 provides an overview), where video footage is not an appropriate approach for understanding experiences for the population involved [20]. Literature describing modifications to traditional touchpoint films are expanding [49], we add our use of still images, summary quotes to convey stakeholder experiences, and modified voice recordings to maintain a consistent rate of speech during playback [27] for supporting collaborative understanding for populations with communication support needs. During individual stakeholder feedback events, PWA and SOs voiced and demonstrated an emotional association with the content presented in the touchpoint film [27]. This suggests, the underlying purpose of creating an emotional connection with the content, remained.

### 4.4 Conclusion

EBCD is a suitable approach for collaborating with PWA and healthcare providers to examine care experiences, identify priorities and codesign areas for change across complex service contexts. While challenges to meaningful engagement exist, flexibility of the EBCD approach supported mitigating modifications, for equitable inclusion. Meaningful involvement of PWA was supported through long‐term engagement, the modified touchpoint film approach, and hybrid methods of data collection. A future direction of the research team will be to implement the findings associated with this body of codesign work. Future research should explore a more detailed evaluation of these processes and their broader application.

## Author Contributions

**Lisa Anemaat:** conceptualisation, investigation, funding acquisition, writing – original draft, methodology, validation, visualisation, writing – review and editing, data curation, project administration, formal analysis. **David A. Copland:** conceptualisation, writing – review and editing, supervision, methodology, funding acquisition. **Victoria J. Palmer:** conceptualisation, writing – review and editing, supervision, methodology. **Sarah J. Wallace:** conceptualisation, writing – review and editing, supervision, methodology, funding acquisition.

## Ethics Statement

Ethical approvals were granted by the Royal Brisbane and Women's Hospital (HREC/2020/QRBW/61368) and The University of Queensland (2020000965) Human Research Ethics Committees.

## Consent

Consent was obtained using standard procedures.

## Conflicts of Interest

The authors declare no conflicts of interest.

## Supporting information

Supplementary Materials.

## Data Availability

The data that support the findings of this study are available on request from the corresponding author. The data are not publicly available due to privacy or ethical restrictions.

## Appendix

See Table A1.

**Table A1:** Overview of participant characteristics contributing feedback across data collection stages.

Participant characteristics (*n* = 127)	PWA (*n* = 32)		SO (*n* = 32)		SP (*n* = 63)	
	*n*	(%)	*n*	(%)	*n*	(%)
Geographic remoteness						
Metropolitan	21	(66)	17	(53)	40	(63)
Regional	8	(25)	12	(38)	17	(27)
Remote	3	(7)	3	(9)	6	(10)
Aphasia severity (PWA/*SO)						
Mild	18	(56)	10	(31)	—	—
Moderate	11	(34)	10	(31)	—	—
Severe	3	(9)	12	(38)	—	—
Time poststroke (PWA/*SO)						
6 weeks–5 months	4	(13)	5	(16)	—	—
6 months–2 years	9	(28)	11	(34)	—	—
2 years–5 years	8	(25)	8	(25)	—	—
5+ years	11	(34)	8	(25)	—	—
Age (PWA/SO)						
18–55 years	8	(25)	11	(34)	—	—
56–70 years	15	(47)	12	(38)	—	—
70+ years	9	(28)	3	(9)	—	—
Years of experience (SP)						
< 2 years	—	—	—	—	7	(11)
2–10 years	—	—	—	—	28	(44)
10+ years	—	—	—	—	28	(44)
Sex						
Female	9	(28)	27	(84)	62	(98)
Male	23	(72)	5	(16)	1	(2)

*Note:* 1: Aphasia severity was determined using the Aphasia Severity Rating Scale (ASRS) on a sample of language collected at time of participation. Severity ratings were assessed independently and confirmed by two speech pathologists (experience gathering phase), for participants recruited after this phase (*n* = 4 PWA), severity was determined according to self‐report of their most recent speech pathologist assessment of language. We did not determine the types of aphasia diagnosed. 2: Remoteness was determined using the Australian Bureau of Statistics Remoteness Area Classifications and were based on post‐codes of site represented. 3: Of the people with aphasia and significant others providing feedback, 11 were born overseas and all spoke English. English was a second language for 7 participants providing feedback. In the main study, interpreters and translators were engaged to support the involvement of one dyad who did not speak English. 4: No student speech pathologists took part, and all were practicing clinically at the time of participation. * of person with aphasia.

Abbreviations: PWA, person with aphasia; SO, significant other; SP, speech pathologist.
