# Supplementary material for: Co‐Designing Aphasia Services: Evaluation of Involvement and Processes to Support Inclusion of People With Post Stroke Aphasia
Source: Health Expect. 2025 Aug 10;28(4):e70372. doi: 10.1111/hex.70372 (PMC12335853; doi:10.1111/hex.70372)
Supplement: Supplementary file 1 — Supplementary Materials. [file HEX-28-e70372-s001.pdf]

Supplementary Materials (SM1-5)

SM1. *Overview of participant involvement across all data collection stages*

**Participant involvement (n=127)**	PWA (n=41)<br>n	**PWA (n=32)<br>n**	SO (n=35)<br>n	**SO (n=32)<br>n**	SP (n=75)<br>n	**SP (n=63*)<br>n**
**Experience gathering (EBCD-2) (n=129)**	Data collection (n=32)	Feedback (n=28)	Data collection (n=30)	Feedback (n=29)	Data collection (n=62^)	Feedback (n=60)
Interview only	*6*	28	*7*	29	*9^*	62^
Focus group	*26*		*23*		*58*	
**Total survey 1 feedback participants (n=117, response rate: 91%)**	***(n=28, R=88%)***		***(n=29, R=97%)***		***(n=60, R=93%)***	
**Prioritisation survey (EBCD-4) (n=82)**<br>*(participants contributing to prior stages: PWA=11; SO=8; SP=38)*	Data collection (n=19)	Feedback<br>-	Data collection (n=12)	Feedback<br>-	Data collection (n=51)	Feedback (n=16)
Survey only	8	-	4	-	13*	16
Survey + prior stages	11		8		38	
**Total survey 2 feedback participants (n=16, response rate: 31%)**	-		-		***(n=16, R=31%)***	
**Consensus groups (EBCD-4-5) (n=18)**<br>*(participants contributing to prior stages: PWA=3; SO=3; SP=10)*	Data collection (n=4)	Feedback (n=1)	Data collection (n=4)	Feedback (n=2)	Data collection (n=10)	Feedback (n=9)
Consensus group 1	4	1	4	2	-	9
Consensus group 2	-		-		10	
Consensus group 3	3		4		3	
**Total survey 3 feedback participants (n=12, response rate: 67%)**	***(n=1, R=25%)***		***(n=2, R=50%)***		***(n=9, R=90%)***	
**Co-design workshops (EBCD-6a) (n=7)**<br>*(all participants contributed to prior stages)*	Data collection (n=1)	Feedback (n=1)	Data collection (n=2)	Feedback (n=2)	Data collection (n=4)	Feedback (n=1)
Workshop 1	1	1	2	2	4	1
Workshop 2	1		2		1	
Workshop 3	1		2		1	
**Total survey 4 feedback participants (n=4, response rate: 57%)**	***(n=1, R=100%)***		***(n=1, R=100%)***		***(n=4, R=25%)***	
**°Consumer Advisory Group (n=6)**	(n=3)	(n=3)	(n=2)	(n=2)	-	-
Duration of project (EBC D 1-6a)	3	3	2	2	-	-
**Total survey 5 feedback participants (n=6, response rate: 100%)**	***(n=3, R=100%)***		***(n=2, R=100%)***		-	

*Details of speech pathologists contributing to survey 2 feedback was not collected to ensure anonymity when responding, therefore overall unique speech pathologists contributing is not able to be accurately calculated (at least 63 unique speech pathologists responded to a feedback survey for at least one activity associated with data collection).

^5 Speech pathologists participated in an interview and a focus group, 2 speech pathologists provided feedback on both their interview and focus group experiences (feedback survey 1).

°Members of the consumer advisory group were not also participants in the research. The sixth member of the consumer group was a cultural capability officer.

PWA=Person with aphasia, SO=Significant other, SP=Speech Pathologist, R=Response rate.

Note: (data collection) refers to the number of unique participants who contributed to each stage of data collection across the research, and (feedback) refers to the proportion of those participants who provided feedback on their experiences of their involvement. Feedback on experiences of involvement is being reported on in this research.

Note: Where possible, participants for subsequent stages were sampled from those who had previously contributed. Additional recruitment was undertaken to support diversity of representation.

SM2. *Example of a 5-point scale and open-ended questions asked in experience survey for people with aphasia during focus group involvement (EBCD stage 2)*

Excerpt from survey 1

Q5. Were you able to communicate what you wanted to share with the group?

Yes – I could communicate **everything** I wanted to	Yes – I said **most things** comments?	Not sure	No – there were **some things** that I didn't say	**No** – I was not able to say anything that I wanted to

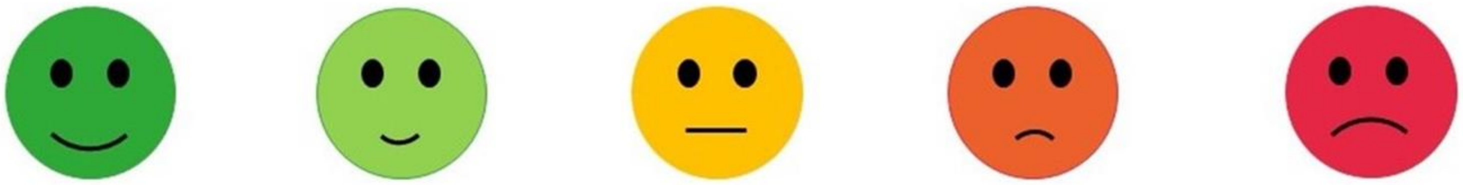

Q9. What could be improved if we ran this group activity again?

Q10. Do you have any other comments?

SM3. *Overview of adaptations made to participant feedback stages and priority identification, to support inclusion.*

Note 1: An overview of the adaptations to the 1) prioritisation procedure; 2) prioritisation survey; 3) touchpoint film; 4) persona development and emotion mapping resources, to support communication for people with aphasia are provided.

Note 2: Accessibility of formatting of content and processes for people with aphasia have been informed by (Anglade et al., 2022; Baker et al., 2021; Brady et al., 2013; Kagan 1998; Pearl and Cruice 2017; Rose et al., 2012; Shiggins et al., 2022) and as presented in Anemaat et al., 2024; Anemaat et al., 2025).

1. Adaptations made to procedure for participant feedback and priority identification for co-design stages during consensus meetings, to support inclusion

1

Procedure:

1. Participants view and reflect on touchpoint film.
2. Review experience maps to understand / answer questions related to experiences of care.
3. Rounds of emotion mapping to select priorities for co-design.

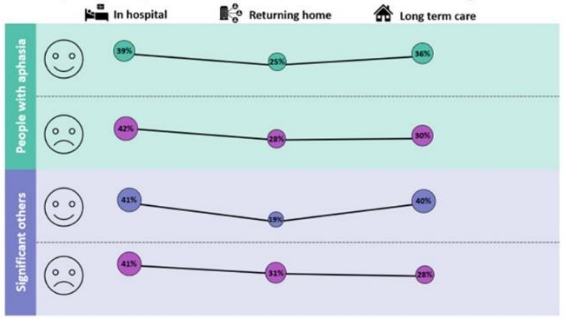

2

Simplified experience maps used to synthesise and support understanding of complex information

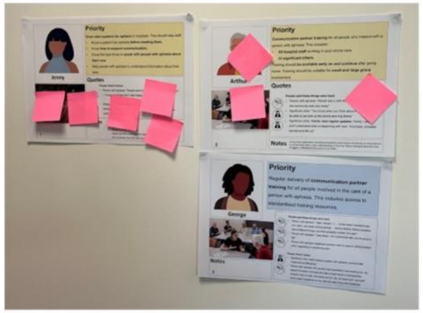

3

Rounds of emotion mapping involved participants placing a coloured sticky-note on the persona they felt the strongest emotional connection with.

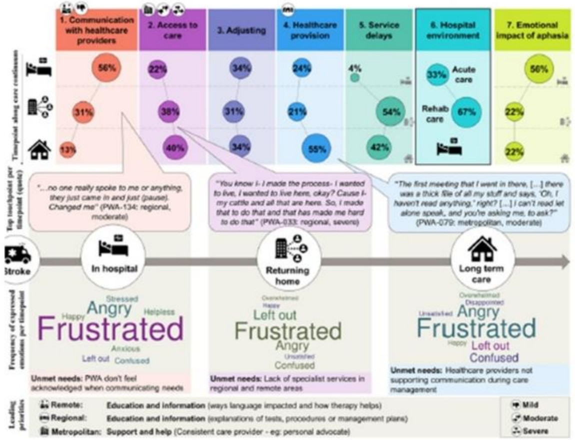

Experience map used to convey touchpoints associated with experiences of care for people with aphasia across the continuum of care (as depicted in Anemaat et al., 2024, *Understanding experiences, unmet needs and priorities related to post-stroke aphasia care: stage one of an experience-based co-design project* BMJ Open).

Emotion mapping: Sticky notes added to personas following a round of emotion mapping. Different personas were placed around the room and those present had time to review and ask clarifying questions prior to making selections. Personas that displayed overlapping priorities identified by different stakeholder groups, were place together, in a vertical line directly above/below each other and seen in the pair on the right in the image above. Full details of consensus workshops have been published elsewhere, including the above image, as seen in: Anemaat L, et al., 2025, *Priorities for post-stroke aphasia service development: An experience-based co-design study.* Clinical Rehabilitation.

2. Prioritisation survey development

Conditional formatting used to limit options for people with aphasia, to reduce cognitive load while making selections. Survey image supports, number of options to select from, pace and volume of audio supports, survey navigation, font size displayed were designed and tested with people with aphasia prior to use in data collection.

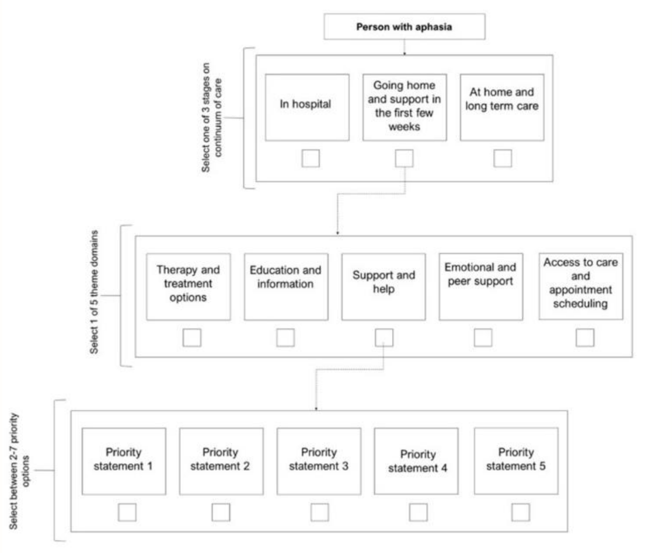

Conditional formatting used to limit options and support navigation

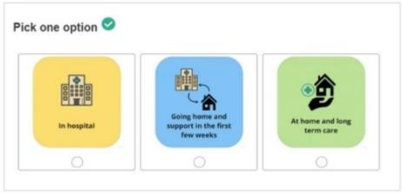

Screen shot of journey of care options to select form

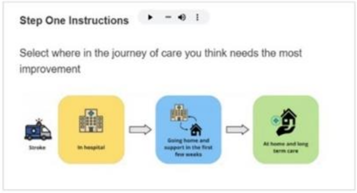

Example of instructions provided

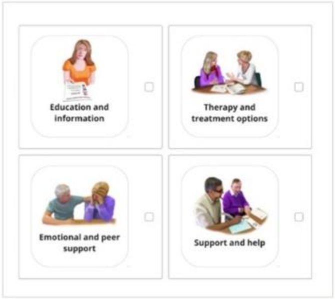

Screen shot of theme domain options to select form

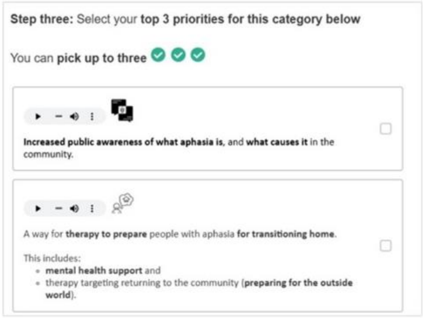

Screen shot of some individual priority options

Examples of aphasia supportive formatting applied

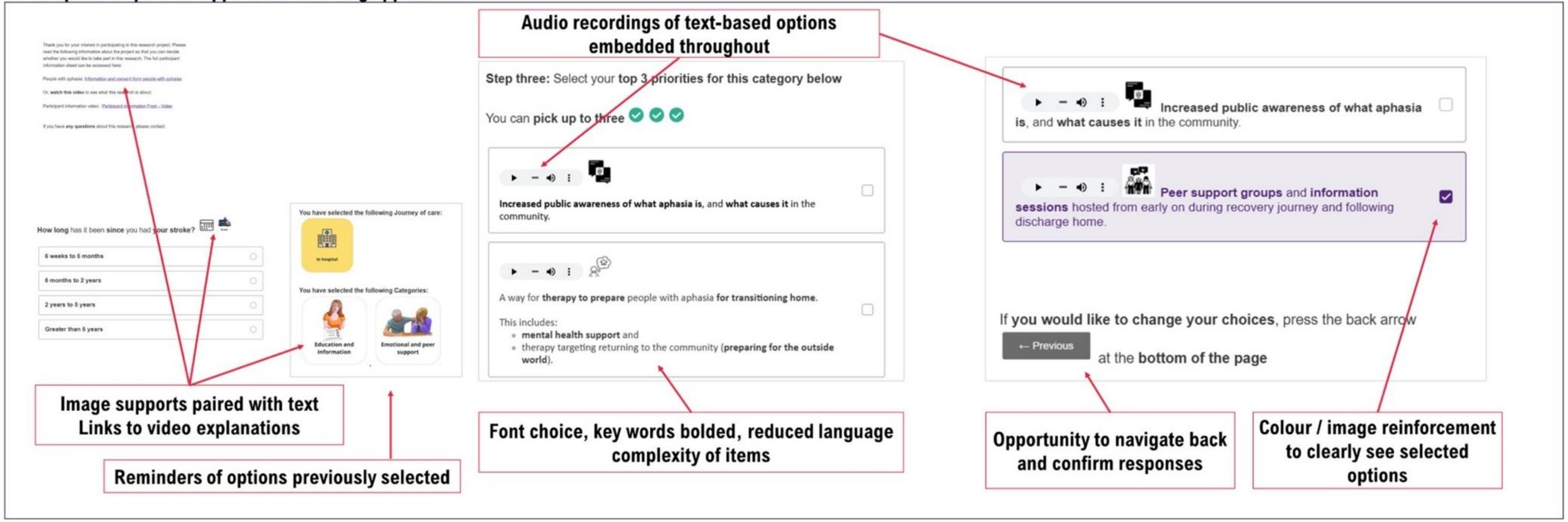

3. Touchpoint film development

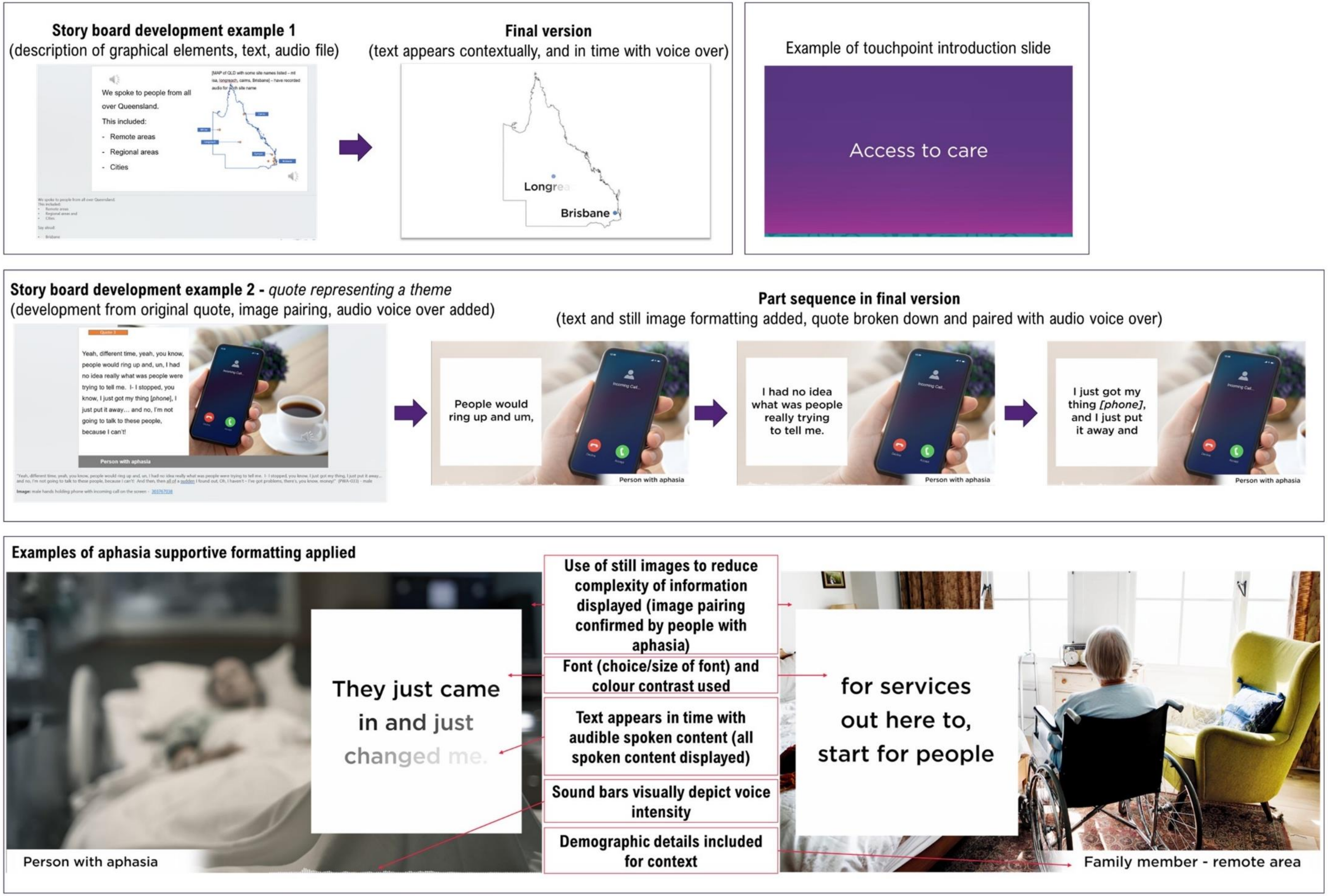

Screen shots taken from: (https://youtu.be/RUpUdITuF1s) – Freely accessible YouTube link to film, available for participants to review at own pace.

Accessibility of formatting of content informed by (Baker et al., 2021; Brady et al., 2013; Kagan 1998; Pearl and Cruice 2017; Rose et al., 2012; Shiggins et al., 2022).

4. Persona development

1. Complexity of priority wording (identified in earlier stages) simplified

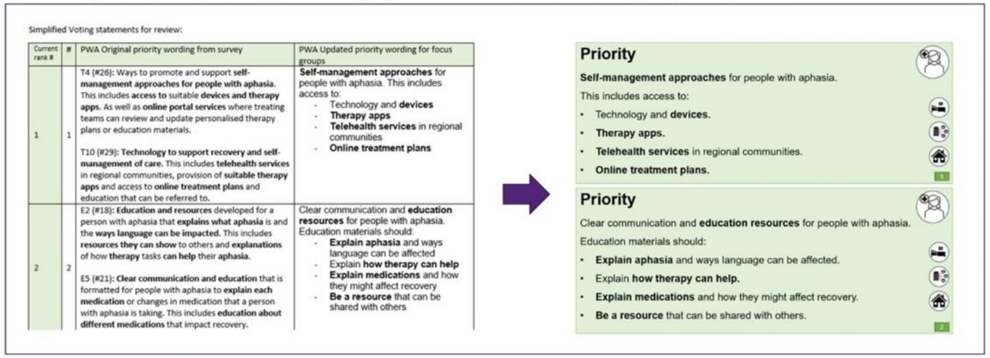

2. Development of resource elements for co-design activity to create personas

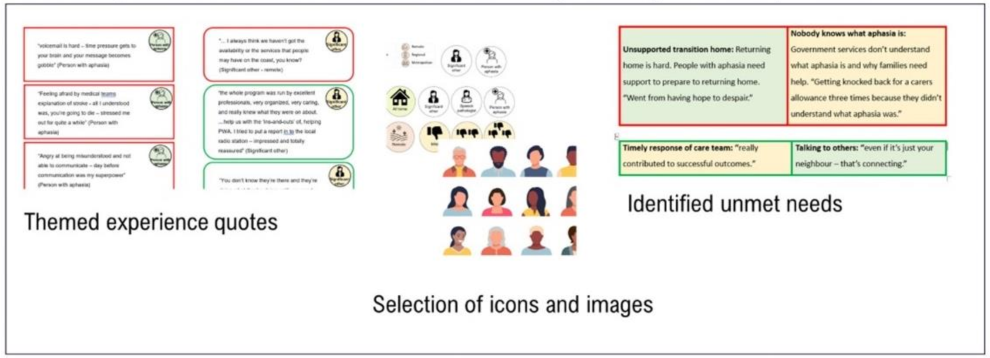

3. Co-designers select image and name for each persona to represent each priority area; 4. card sort activities to pair experience quotes and unmet needs; 5. Notes and additional images added to support understanding

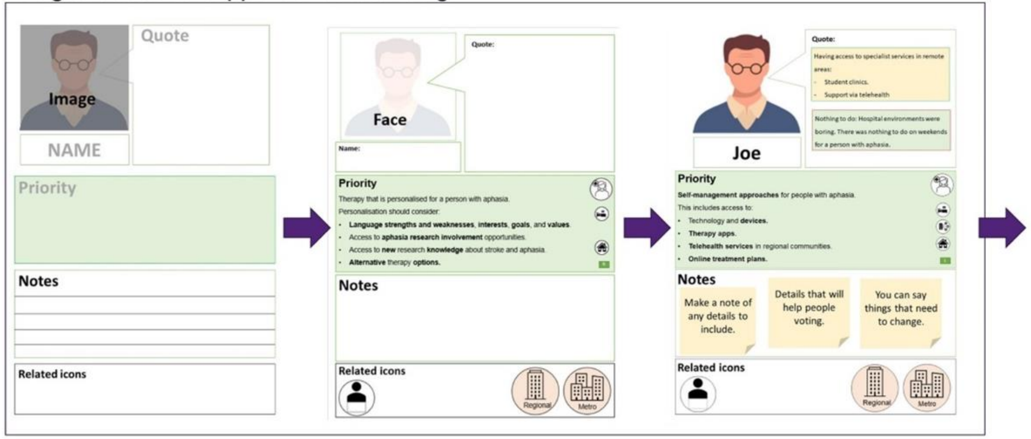

Examples of aphasia supportive formatting applied

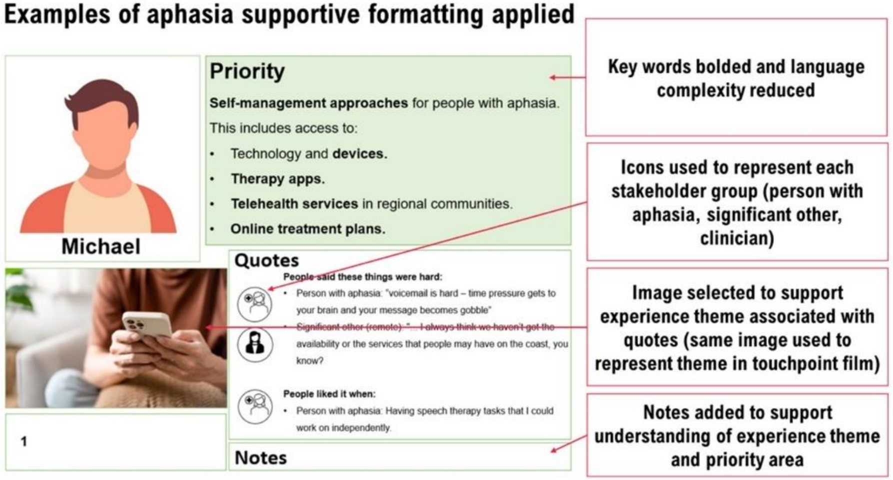

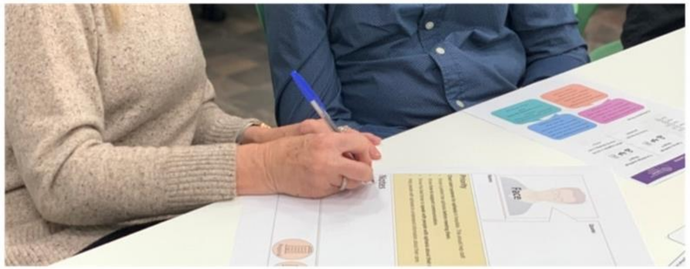

Co-designers adding notes and comments to persona template.

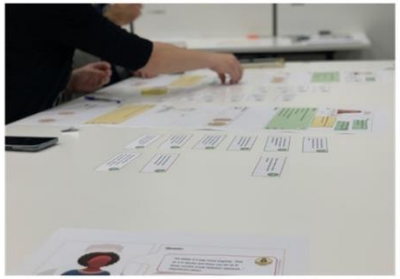

Co-designers pairing quotes with priority area

SM4. *Comparative overview of different timelines of involvement across stakeholder groups.*

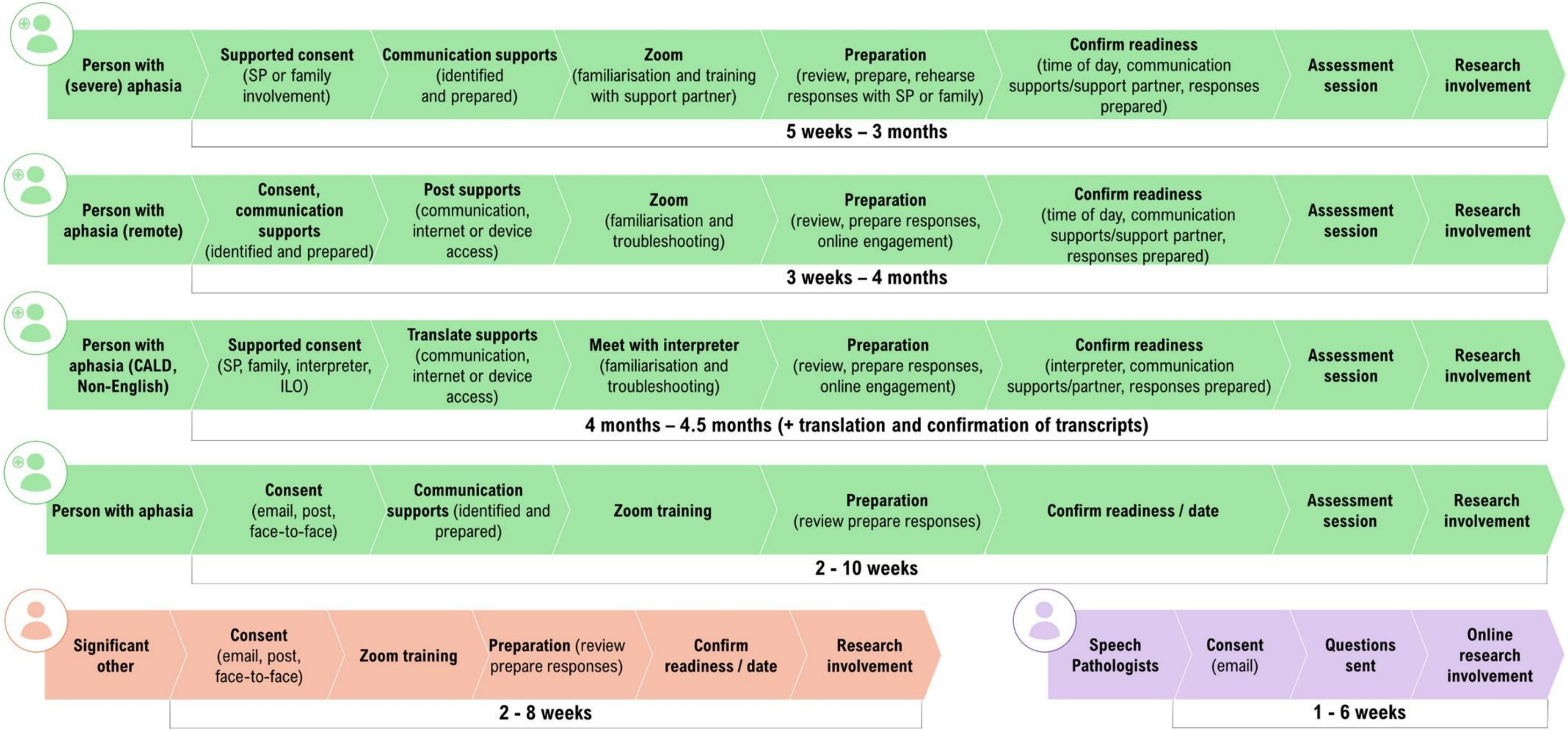

CALD=Culturally and linguistically diverse

**Some online assessment sessions were not completed (assessments were conducted by treating speech pathologist with interpreter present).*

Note: shorter time frames where people with aphasia were involved, included a hybrid approach to recruitment (face to face discussion of project, informed consent and provision of project information, prior to online practice sessions, assessment and research involvement).

SM5. Engagement of people with aphasia across work presented, according to the PAOLI Frameworks items and statements for guiding public involvement in aphasia research

Phase and item	Details of involvement	Reported on page #
Phase 1: Foundation		
1: Establishing collaborations	- Contact was made with 43 local aphasia groups, and 3 national networks [25]<br>- This research partnered with 21 hospital and health service sites [26]	Refs 25-27<br>Pg 11, 15-16<br>Figure 2
2: Recruitment	- Recruitment video and tailored recruitment information (culturally respectful recruitment flyers) were developed with the consumer advisory group [16]<br>- Consumer advisory group member contributions (figure 2)	Ref 16<br>Figure 2<br>Pg 4--6
3: Gaining informed consent	- Consent forms were designed in an accessible format [25-27]	Ref 25-27<br>Pg 12-13, 15
4: Induction	- SM3 shows recruitment timelines for those contributing to the research<br>- Details of consumer advisory group contributions (figure 2)	Pg 12-13,15<br>Figure 2<br>SM3
5: Patient / partner training	- All public participants received training in methods prior to engagement<br>- All participants with aphasia selected mode of participation and identified most appropriate communication supports to facilitate engagement [25,27]<br>- SM3 details comparative timelines of involvement for supporting people with aphasia across aphasia severities (ASRS ratings 1-5) [25]	Pg 12-13,15<br>SM3
6: Creating communication links	- Communication accessible formatting was used across all engagement methods [16,25-27]	Pg 12-13, 15<br>Refs 16,25-27<br>Figure 2
7: Engaging communication partners	- All participants with aphasia selected mode of participation and identified most appropriate communication supports to facilitate engagement [25,27]	Ref 25, 27<br>Figure 2<br>Pg 12-13, 15
Phase 2: Development		
8: Conceptualisation	- Consumer advisory group members contributed to the design of all research elements (figure 2) [16,25-27]<br>- Experience gathering stages of the research involved interviews and focus groups to explore experiences, determine ideas for change and identify local priorities for change [25]	Pg 5,12-15<br>Refs 16,25-27<br>Figure 2
9: Establishing research priorities	- Experience gathering stages of the research involved interviews and focus groups to explore experiences, determine ideas for change and identify local priorities for change [25]<br>- Priorities for change were collaboratively agreed by stakeholders [27]	Ref 25,27<br>Figure 2<br>Pg 5
10: Reaching consensus	- Experience gathering stages of the research involved interviews and focus groups to explore experiences, determine ideas for change and identify local priorities for change [25-27]<br>- Priorities for change were collaboratively agreed by stakeholders [27]	Refs 25-27<br>Figure 2<br>Pg 5-6, 20-21
11: Co-design methodology	- Consumer advisory group members contributed to co-designing and testing of all methods of data collection [16,25-27] (figure 2)<br>- Clear instructions and resources, suitably formatted, were sent ahead of time so participants had time to process information.<br>- Roles, responsibilities and an overview of tasks were provided prior to and at the start of each meeting	Refs 16,25-27<br>Figure 2<br>Pg 5-6, 12-13, 17-18

12: Proposal development	- People with aphasia who were members of the consumer advisory group were co-authors on all papers [16,25-27]<br>- Consumer advisory group members contributed across all phases of the research, described in figure 2	Refs 16,25-27<br>Figure 2<br>Pg 5, 12-13
Phase 3: Translational		
13: Outcomes and implementation	- People with aphasia who were members of the consumer advisory group were co-authors on all papers [16,25-27]<br>- Consumer advisory group members contributed across all phases of the research, described in figure 2, including as co-presenters at conferences	Refs 16,25-27<br>Figure 2<br>Pg 5, 12-13
14: Dissemination and sustainability	- People with aphasia who were members of the consumer advisory group were co-authors on all papers [16,25-27]<br>- Consumer advisory group members contributed across all phases of the research, described in figure 2, including as co-presenters at conferences	Refs 16,25-27<br>Figure 2<br>Pg 5, 12-13
Phase 4: Ongoing processes		
15: Support and self-evaluation	- Consumer advisory group members evaluated their contributions to the research.	Pg 6, 10
16: Monitoring	- An updates and reports on the research have been co-designed with members of the consumer advisory group (presented as suitably formatted video abstracts) for community audiences, and are all freely available via youtube and linked to in published findings [25-27]	Refs 25-27
17: Impact	- Personal reflexive critique acknowledges the positive impact of our public partners had on research processes, recruitment and dissemination.	Pg 4-6, 10-18
PAOLI Framework: *Charalambous, M., Kountouri, A., Schwyter, J.R. et al. The development of the People with Aphasia and Other Layperson Involvement (PAOLI) framework for guiding patient and public involvement (PPI) in aphasia research. Res Involv Engagem 9, 74 (2023). https://doi.org/10.1186/s40900-023-00484-9.*		

Note: ASRS=Aphasia Severity Rating Scale (Goodglass H, Kaplan E, Barresi B, Weintraub S. Boston diagnostic aphasia examination. 3rd ed. ed. Philadelphia: Lippincott Williams & Wilkins; 2001.)

Journey Map Example

	1	2	3	4
**Point of Stroke**	*ACUTE – early diagnosis & ax stabilisation of condition / acute (medically unstable) – 0-1mnth*	*SUBACUTE - medically stable - greatest spontaneous recovery + REHAB - in-pt (1-4mnths)*	*SUBACUTE - medically stable - greatest spontaneous recovery + REHAB (Community based - 4-9mnths)*	*CHRONIC - ongoing recovery and rehabilitation 9+mnths*
	Barriers	***Barriers***	***Barriers***	***Barriers***
	**Pt factors** (general health and stability, mood, motivation, attitude) **Environmental factors** (eg: from a RACF or from home with support person) **Funding barriers** (as per stroke pathway - ax within 24hrs) **Hospital barriers** (eg/ physical space, beds and resourcing - eg: code yellow (at capacity)), **Staffing** (generalist staffing, managing multiple caseloads) - Funding (incentives) helps motivate / (designated ASU-acute Stroke unit).	**Pt factors** (Dependent on MDT input- if independently mobile - access to rehab is reduced - general health and stability, mood, motivation, attitude) **Environmental factors** (eg: from a RACF or from home with support person). **Funding barriers** (pt coding - QH/health condition). **Hospital barriers** (eg/ physical space, beds and resourcing - eg: code yellow (at capacity) - Allocation of resources - when hospital is at capacity staff are reassigned to cover acute. Rehab spaces doesn't have internet access to support service delivery - eg: apps on ipads), **Staffing** (generalist staffing, managing multiple caseloads).	**Pt factors** (general health and stability, mood, motivation, attitude) **Environmental factors** (eg: home with support person). **Funding barriers** (Time limited service - 12 wks - not EBP - based on service directive - eligibility for NDIS / My Aged Care). **Hospital barriers** (transport to in-pt / clinic setting) **Staffing** (funding / FTE for community services).	Severe shortage of community providers to community-based services following d/c from hospital funded care. Extended wait times. Need to self - manage. No local support group. Have funding - but reduced access to the provider (community SP). - Fend for yourself situation.
**Done well**	Able to deliver service in line with service management guidelines	Able to deliver service in line with service management guidelines	Able to deliver service in line with service management guidelines	This is evolving - not currently done well
**Not so well**	Meets needs well - communication across pathway to other services.	Reduced prioritisation (comparatively to other clinical areas (pt flow)) – sometimes moved on too early.	Preparing for d/c - transition from hospital-based services to other services.	Assisting pts to navigate suitable services and systems (Access).
**Confidence Rating 1-10 1= Low 10= High**	**10**	**10**	**10**	**8**

SM6. *Example of aphasia service map following interview with one site*
